# Novltex: A New Class of Antibiotics with Potent Activity against Multidrug-Resistant Bacterial Pathogens—Design, Synthesis, and Biological Evaluation

**DOI:** 10.1021/acs.jmedchem.5c01193

**Published:** 2025-09-16

**Authors:** Esra Malkawi, Anish Parmar, Sanjit Das, Enas Newire, Charlotte M. Jones, Kate A. Morrison, Milandip Karak, Frédéric Blanc, Nicholas Harper, Rajamani Lakshminarayanan, Zhi Sheng Poh, Navin K. Verma, Jennifer Unsworth, Dallas E. Hughes, Losee Lucy Ling, Stephen A. Cochrane, William Hope, Ishwar Singh

**Affiliations:** † Antimicrobial Pharmacodynamics and Therapeutics, Department of Pharmacology and Therapeutics, William Henry Duncan Building, 4591 University of Liverpool, Liverpool L7 8TX, U.K.; ‡ Department of Chemistry, University of Liverpool, Liverpool L69 7ZD, U.K.; § College of Pharmacy, Amman Arab University, Amman 11953, Jordan; ∥ School of Chemistry and Chemical Engineering, 1596 Queen’s University Belfast, David Keir Building, Stranmillis Road, Belfast BT9 5AG, U.K.; ⊥ Leverhulme Research Centre for Functional Materials Design, Materials Innovation Factory, University of Liverpool, Liverpool L7 3NY, U.K.; # Stephenson Institute for Renewable Energy, University of Liverpool, Liverpool L69 7ZF, U.K.; ¶ Singapore Eye Research Institute, The Academia, Discovery Tower Level 6, 20 College Road, Singapore, 169857, Singapore; ∇ Department of Pharmacy & Pharmaceutical Sciences, National University of Singapore, Singapore, 117543, Singapore; ○ Ophthalmology and Visual Sciences Academic Clinical Program, Duke-NUS Graduate Medical School, Singapore, 169857, Singapore; ⧫ Lee Kong Chian School of Medicine, 54761 Nanyang Technological University Singapore, 11 Mandalay Road, 308232, Singapore; †† NovoBiotic Pharmaceuticals, Cambridge, Massachusetts 02138, United States

## Abstract

Increasing spread of multidrug-resistant (MDR) bacteria demands antibiotics that combine potent activity with scalable synthesis. Novo29 (clovibactin) is promising but suffers from low yield (1%), dependence on costly and noncommercial d-hydroxy-asparagine (d-Hyn_5_), and lengthy syntheses. We report “Novltex”, a novel class of antibiotic that fuses the Leu_10_-teixobactin macrocycle to the Novo29 N-terminus tail, replacing d-Hyn_5_ with inexpensive threonine. Our efficient synthesis delivers 30% yield with faster coupling cycles (∼10 min), enabling rapid and low-cost scale-up. A 16-member analogue library systematically probing amino-acid configuration identified analogue **4** (d-Leu_2_) as the initial lead, informing the rational design of analogue **12** (d-cyclohexylalanine_2_). Analogue 12 displays potent antibacterial activity (minimum inhibitory concentration (MIC) 0.12–0.5 μg/mL) against World Health Organization (WHO)-priority pathogens, including methicillin-resistant *Staphylococcus aureus (*MRSA) and *Enterococcus faecium*, surpassing several licensed antibiotics while maintaining an excellent safety profile. Lipid II-binding assays confirm the conservation of the parent mechanism. Novltex, therefore, offers a practical, high-yielding, and cost-efficient platform for the development of next-generation antibiotics targeting MDR infections.

## Graphical abstract

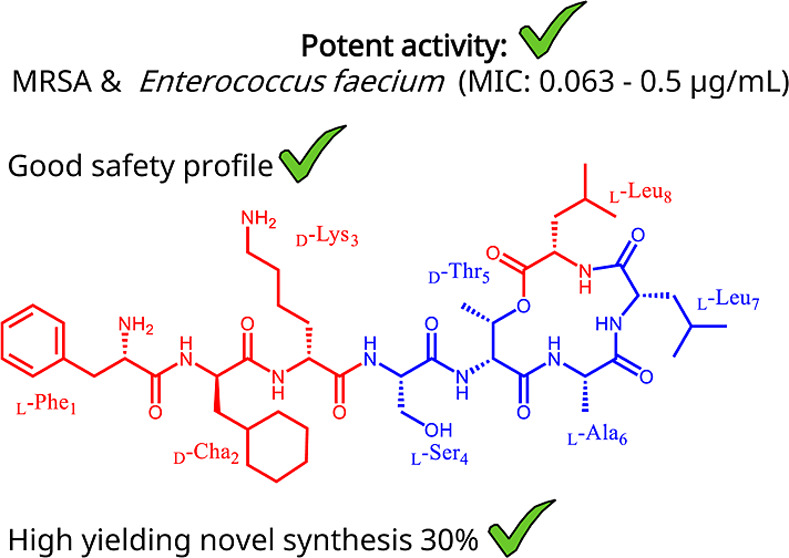

## Introduction

Antimicrobial resistance (AMR) is a critical global health threat, driven by rising resistance to existing antibiotic classes and lack of new classes of antibiotics to address this urgent medical need.^1,2^ In 2019, antibiotic-resistant infections were associated with approximately 5 million deaths worldwide.^3^ This challenge is further exacerbated by a lack of innovation in the antibiotic pipeline, underscoring the urgent need for the discovery and development of new antibiotic classes to tackle AMR.^1^ Although AMR is a global issue, its impact is disproportionately higher in low- and middle-income countries (LMICs).^3^ This highlights the necessity for therapeutic solutions that are both effective and accessible in diverse clinical settings including LMICs. To address this, we and others have explored Arg_10_-teixobactin and its analogues as simplified derivatives of natural teixobactin, a promising new antibiotic class.^4–9^ Further advancements have led to counterintuitive designs aimed at improving potency, safety, and cost-effectiveness.^10–13^ Notably, we replaced the challenging and costly enduracididine (a cationic side chain) with hydrophobic, noncharged residues such as leucine (Leu_10_-teixobactin), resulting in enhanced potency.^10–13^

Novo29, a newly discovered class of antibiotic (Figure 1A), was identified from β-proteobacteria by NovoBiotic Pharmaceuticals.^14^ Its macrocyclic ring shares structural similarities with Leu_10_-teixobactin (Figure 1B). Notably, Novo29 also features a leucine residue at the same position within the macrocycle as Leu_10_-teixobactin,^10^ though the numbering differs due to variations in peptide length (Figure 1). Structurally, Novo29 is a cyclic depsipeptide containing a 13-membered macrocycle (Figure 1A). Although Novo29 was initially described as one of 11 compounds classified under formula (III),^14^ its configurational details remained unclear, and no experimental validation was provided. To address these gaps, we developed a novel design and an efficient synthetic approach. For clarity, we selected a representative molecule (Figure 1A) from formula (III), as reported by Novobiotic, for our studies.

**Figure 1:**
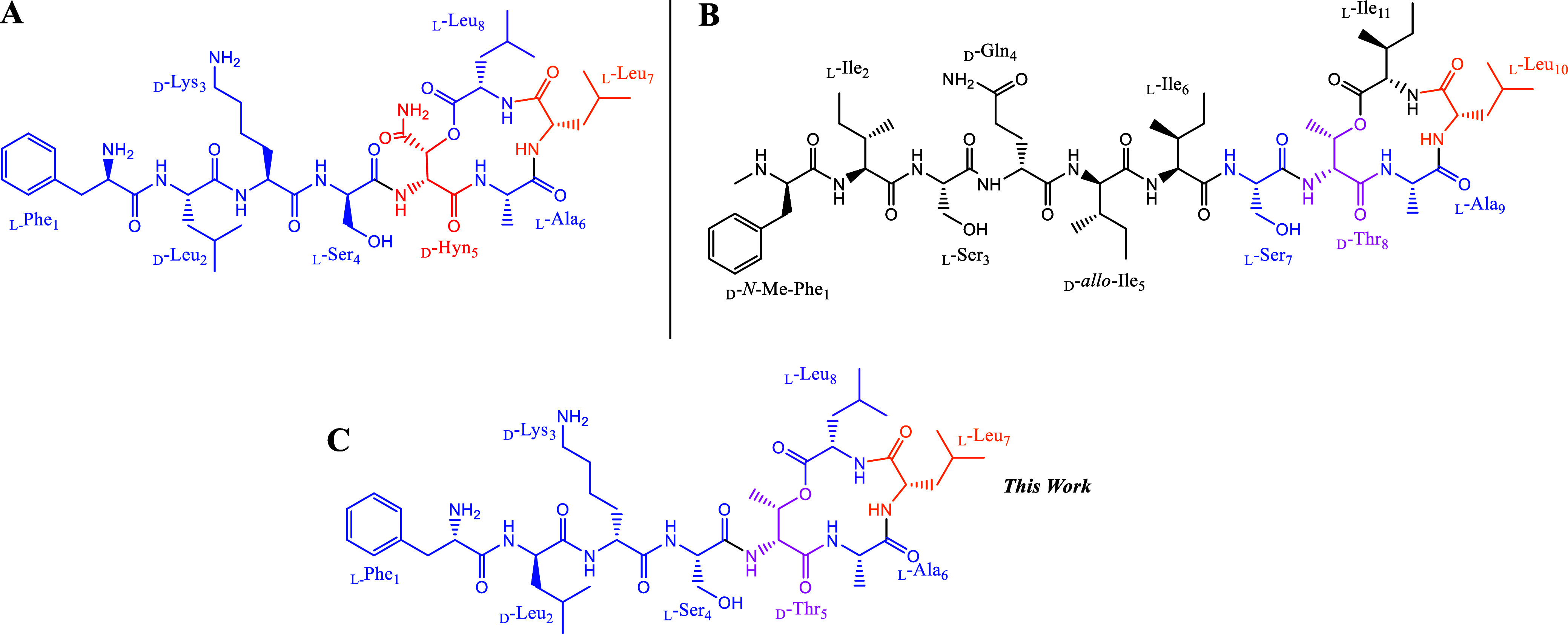
(A) Novo29/clovibactin. (B) Leu_10_-teixobactin. (C) Novltex analogue **4** (colors show residues inherited from (A) or (B)).

Novo29 is an octapeptide antibiotic composed of four l-amino acids (Phe_1_, Ser_4_, Ala_6_, and Leu_8_), three d-amino acids (Leu_2_, Lys_3_), and an unnatural (2*S*/*R*, 3*R*) d-hydroxy asparagine (d-Hyn_5_), which serves as the connection point between the macrocycle and the tail. The stereochemical configuration and total synthesis of Novo29 were later reported by Nowick et al.,^15^ while Weingarth et al.^16^ provided comprehensive biological characterization and mode-of-action studies on Novo29, also referred to as clovibactin.

Novo29 exerts its antibacterial activity by simultaneously targeting the pyrophosphate moieties of multiple essential peptidoglycan precursors (C_55_-PP, lipid II, and lipid III), effectively killing drug-resistant Gram-positive bacteria without inducing resistance.^16^ These targets are highly conserved across bacterial species and absent in mammalian cells, making them highly desirable for antibiotic development.

The reported synthesis of Novo29 is highly challenging and suffers from a low yield (∼1%). Additionally, it relies on the incorporation of a unique, noncommercially available building block, d-Hyn_5_, further restricting its practical use. The inclusion of d-Hyn_5_ significantly increases production costs and presents synthetic challenges, as its preparation requires a complex multistep synthesis.^17^ Nowick et al.^17^ reported a structure–activity relationship (SAR) study on Novo29 and its analogues, providing further insights into its bioactivity.

As part of our ongoing efforts to simplify natural products to enhance safety, efficacy, and accessibility while reducing costs, we hypothesized that integrating the macrocycle of Leu_10_-teixobactin (which utilizes the commercially available, low-cost threonine instead of the synthetically challenging d-Hyn_5_) with the tail of Novo29 could provide a viable solution to overcome Novo29’s limitations.

To test this hypothesis, we synthesized a series of hybrid antibiotics, which we named “Novltex” (Figure 1C). Additionally, we investigated the impact of amino acid configuration on antibacterial activity to identify optimal candidates. A position 2 scan of Novltex further led to the discovery of Novltex analogue **12**, a highly potent molecule effective against multidrug-resistant (MDR) bacterial pathogens, including methicillin-resistant *Staphylococcus aureus* (MRSA) and *Enterococcus faecium*, priority pathogens recognized by the World Health Organization (WHO) due to their rising mortality rates and significant healthcare burden.^18^

## Results and Discussion

### Design and Synthesis

We developed an efficient, high-yielding novel synthetic approach for Novltex analogues, leveraging commercially available building blocks and rapid microwave-assisted couplings (Scheme 1, Figure S1, Section III, Supporting Information). This methodology enables solid-phase peptide synthesis (SPPS) of Novltex analogues, followed by a single purification step postfinal cleavage, streamlining the overall process and enhancing synthetic efficiency.

**Scheme 1:**
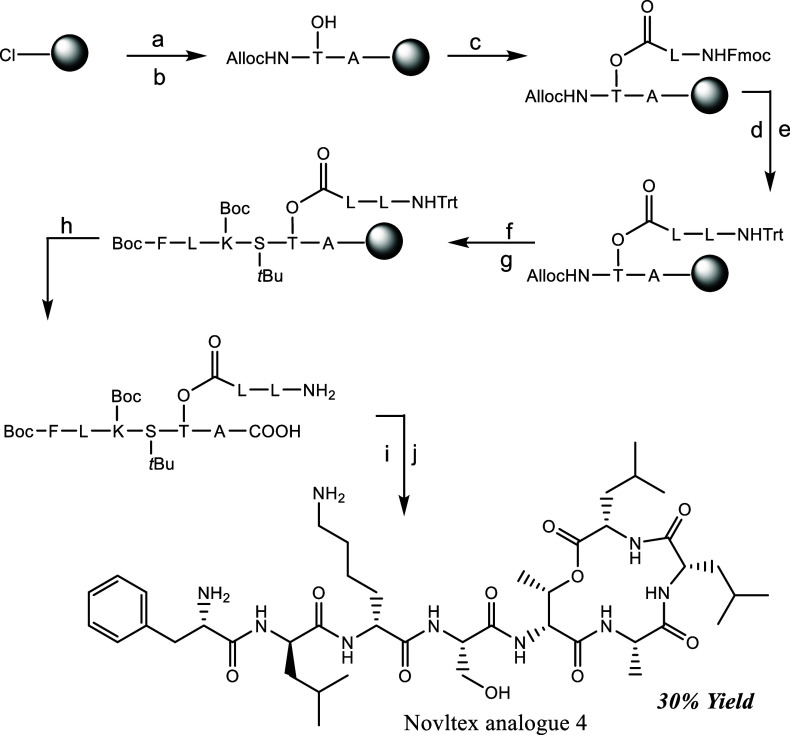
Total Synthesis of Novltex Analogue **4** Starting from 2-Chlorotritylchloride Resin: (a) 4 eq. of Fmoc-Ala-OH/8 eq. of DIPEA in DCM, 3 h. (b) 20% Piperidine in DMF Followed by 3 eq. of AllocHN-d-Thr-OH and 3 eq. of HATU/6 eq. of DIPEA. (c) 10 eq. of Fmoc-Leu-OH, 10 eq. of DIC, 5 mol % DMAP in DCM, 1 h followed by Capping with Ac_2_O/DIPEA 10% in DMF, after with 20% Piperidine in DMF. (d) 4 eq. of Fmoc-Leu-OH, 4 eq. of HATU/8 eq. of DIPEA in DMF, 1 h Followed by 20% Piperidine in DMF. (e) 10 eq. of Trt-Cl, 15% Et_3_N in DCM, 1 h. (f) 0.2 eq. of [Pd­(PPh_3_)_4_]^0^ /24 eq. of PhSiH_3_ in dry DCM, 1 × 30 min, 1 × 45 min. (g) 4 eq. of Fmoc/Boc-AA­(PG)–OH (AA = Amino Acid, PG = Protecting Group), 4 eq. of DIC/Oxyma (μwave, 10 min) Followed by 20% Piperidine in DMF (μwave 3 min, RT, 10 min). (h) TFA: TIS: DCM = 2:5:93, 1 h. (i) 1 eq. of HATU/10 eq. of DIPEA in DMF, 30 min. (j) TFA: TIS: H_2_O = 95:2.5:2.5, 1 h

We synthesized the Novltex analogues by loading Fmoc-alanine onto 2-chlorotrityl chloride resin, followed by an amide coupling with Alloc–NH–d-Thr-OH (Scheme 1). Esterification was performed with Fmoc-Leu-OH or Fmoc-Ile-OH with DIC and 5 mol % DMAP for 1 h, followed by coupling the next amino acid using HATU and DIPEA in DMF. Fmoc was deprotected, and the amino group was subsequently protected with a trityl. The *N*-terminal alloc protecting group was removed by using [Pd­(PPh_3_)_4_]^0^ and phenyl silane. All other amino acids were coupled using 4 eq. of AA with 4 eq. of DIC/Oxyma using an automated microwave peptide synthesizer (coupling time of 10 min each). Fmoc deprotection was performed with 20% piperidine in DMF: 3 min under microwave irradiation, followed by 10 min at room temperature. Partial cleavage was done using TFA/TIS/DCM (2:5:93), followed by a cyclization using HATU/DIPEA for 30 min. The peptides were then fully cleaved and purified, resulting in high yields between 25 and 30% (Scheme 1). High-performance liquid chromatography (HPLC) and mass spectrometry (MS) data for all analogues and ^1^H and ^13^C NMR data of the representative Novltex analogue **4** confirm the successful synthesis of the molecules in high purity (Figures S3–S42, Sections V and VI, Supporting Information).

### Antibacterial Studies

A total of 10 Novltex analogues (Table 1, Figure S2, and Table S1 and S2, Sections IV and V, Supporting Information) were synthesized to investigate the impact of amino acid stereochemistry in both the tail and macrocycle on antibacterial activity.

**Table 1:**
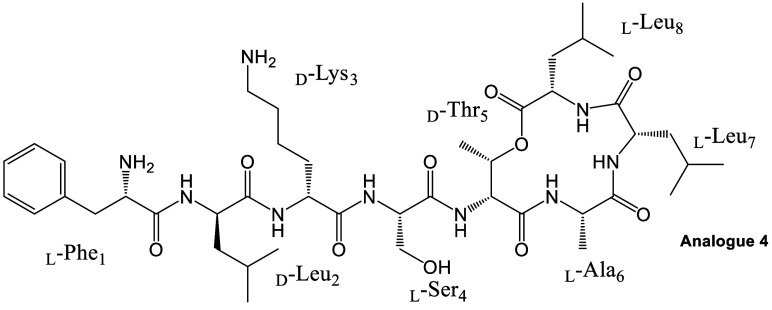
Minimum Inhibitory Concentration (MIC) of the Novltex Analogues (**1**–**10**) against MRSA along with Their Configuration

	analogues configuration								
synthesized Novltex analogues	Phe_1_	Leu_2_	Lys_3_	Ser_4_	Thr_5_	Ala_6_	Leu_7_	Leu_8_/Ile_8_ ^a^	MIC against MRSA ATCC 33591 (μg/mL)
**1**	L	D	D	L	L	L	D	L	8
**2**	L	D	D	L	D	L	D	L	32
**3**	D	L	L	D	D	L	L	L	32
**4**	**L**	**D**	**D**	**L**	**D**	**L**	**L**	**L**	**2–4**
**5**	D	L	D	L	D	L	L	L	>32
**6**	L	D	L	D	D	L	L	L	>32
**7**	D	D	L	L	D	L	L	L	32
**8**	L	L	L	L	D	L	L	L	>32
**9**	D	D	D	D	D	L	L	L	16
**10**	L	D	D	L	D	L	L	L^a^	8
vancomycin									0.5–1

a Indicates the presence of l-Ile_8_ for analogue **10**.

Among the ten Novltex analogues tested, three demonstrated promising activity against MRSA. Notably, analogue **4** exhibited the highest potency, with a minimum inhibitory concentration (MIC) of 2–4 μg/mL, comparable with the clinically used antibiotic vancomycin. The remaining analogues displayed lower activity, with three showing no detectable antibacterial effect at the highest tested concentration (MIC of ≥32 μg/mL, Table 1).

The amino acid composition of Novltex analogues given in Table 1 is similar to Novo29 (except d-Hyn_5_ is replaced by D/LThr, Novltex analogue **10**, and l-Leu_8_ is replaced with l-Ile_8_, Figure S2). We synthesized the Novltex analogues using different configurations of amino acids (Table 1) to evaluate their role in antibacterial properties against MRSA and to identify suitable combinations for further development. The MIC of Novltex analogue **1** (MIC 8 μg/mL) shows moderate activity against MRSA, indicating that the L configuration at position Thr_5_ and the D configuration at position Leu_7_ were tolerated. However, D configurations at positions Thr_5_ and Leu_7_ together were not tolerated (analogue **2**, MIC = 32 μg/mL).

For analogue **3**, we kept a macrocycle configuration similar to Leu_10_-teixobactin and used the **D**_**1**_ **LLD**_**4**_ configuration for the tail, but this combination was not tolerated (MIC = 32 μg/mL). This indicates that not only position 5 but also the right configurational pairing of amino acids at positions 1, 2, 3, and 4 are crucial for antibacterial activity.

To design Novltex analogue **4**, we adopted the configurations of the macrocycle from Leu_10_-teixobactin^10^ while keeping the tail configurations **L**_**1**_ **DDL**_**4**_. This resulted in a significant improvement in the antibacterial activity of Novltex analogue **4** (MIC = 4 μg/mL) compared to analogues **2** and **3**. This result supports the mode of action studies that have the side-chain orientation of two adjacent hydrophobic residues (Phe_1_ and Leu_2_) on the same side likely to improve membrane interactions and the hydrophilic residues on the opposite side while likely engaging in noncovalent interactions with target lipids.^16^

The remaining analogues (Novltex **5**, **6**, **7**, and **8**) did not show good antibacterial activity (MIC >32 μg/mL), and these outcomes reinforce our hypothesis that having an appropriate configurational arrangement for amino acids in the peptide sequence is important for antibacterial activity. Interestingly, analogue **9** with the **D**_**1**_ **DDD**_**4**_ configuration was tolerated (MIC 16 μg/mL), but it was less potent than analogue **4**. The replacement of Leu_8_ with Ile_8_ is tolerated and shows moderate antibacterial activity (analogue **10**, MIC 8 μg/mL).

Mode-of-action studies on Novo29 suggest that the Leu_2_ side chain interacts with the bacterial membrane.^16^ Building on our efficient synthesis and structural insights into Novltex analogue **4**, we hypothesized that modifying Leu_2_ with hydrophobic residues could enhance membrane interactions, potentially improving antibacterial activity.

To test this, we synthesized a new series of Novltex analogues, incorporating commercially available amino acids with cyclic hydrophobic side chains, including cyclohexyl glycine (Chg), cyclohexyl alanine (Cha), tryptophan (Trp), phenylalanine (Phe), and tyrosine (Tyr), along with the noncyclic hydrophobic amino acid d-*allo*-isoleucine (d-*allo*-Ile) (Figure 2, Figure S2, Tables 2 and S2, Sections IV and V, Supporting Information).

**Figure 2:**
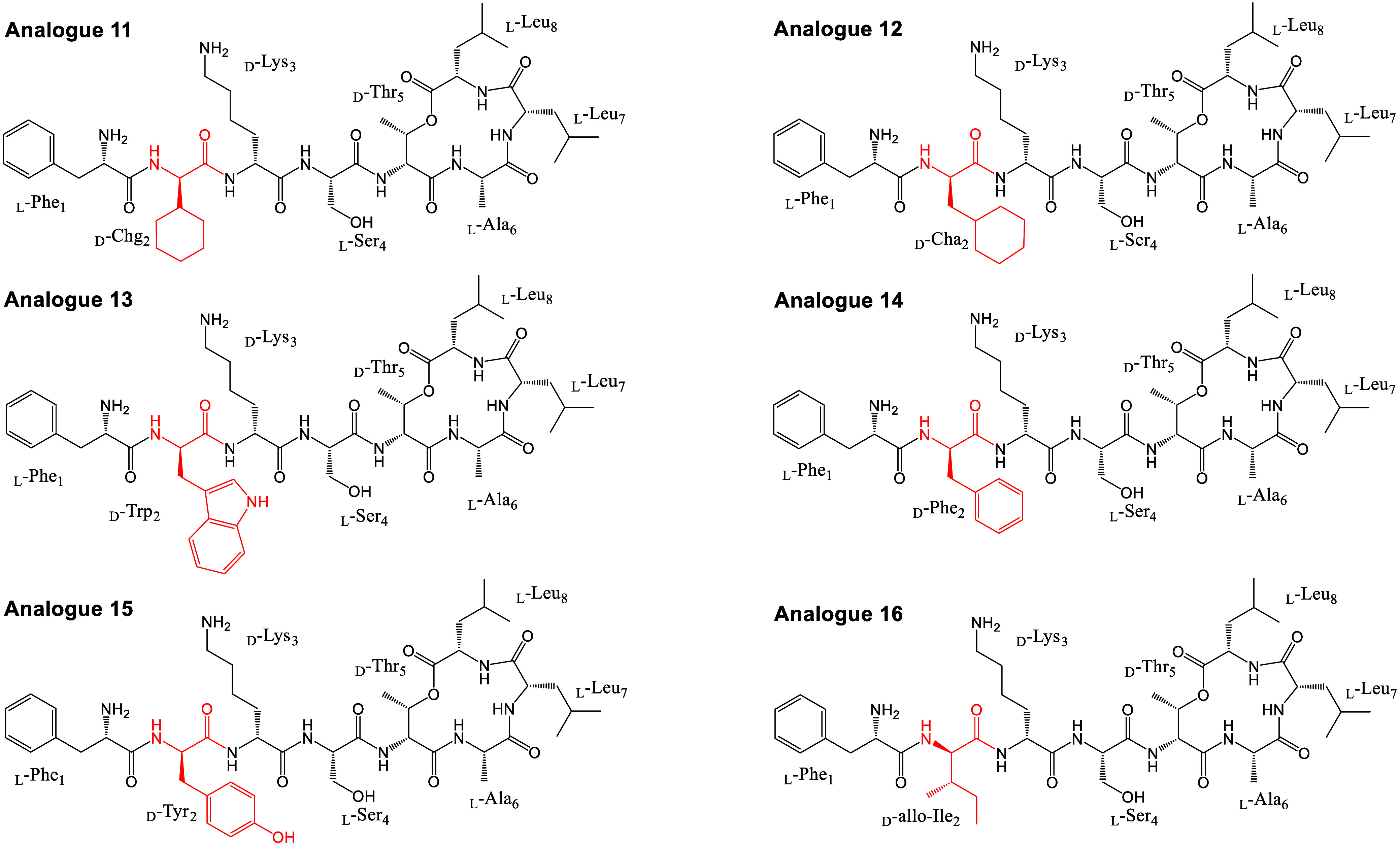
Structures of analogues **11–16.** Analogues are based on the configuration of analogue **4**, and Leu_2_ is replaced with amino acids in red.

**Table 2:** Minimum Inhibitory Concentration (MIC) Values of the Synthesized Novltex Analogues (**11**–**16**) against MRSA^a^

**synthesized Novltex analogues**	**name/composition**	MIC against MRSA ATCC 33591
11	Leu_2_Chg–Novltex	8
**12**	**Leu_2_Cha–Novltex**	**0.25**
13	Leu_2_Trp–Novltex	>32
14	Leu_2_Phe–Novltex	8
15	Leu_2_Tyr–Novltex	>32
16	Leu_2_ d-*allo*-Ile–Novltex	16

a All compounds have the same configuration based on analogue **4**.

The antibacterial activity of the analogues (**11–16**) was tested against MRSA ATCC 33591 (Table 2). Among the Leu_2_ substitutions, analogue **12** with cyclohexyl alanine (Leu_2_Cha) showed potent antibacterial activity with a strikingly low MIC value of 0.25 μg/mL compared to analogue **4**. Interestingly, analogue **11**, which also contains a cyclohexyl group (cyclohexyl glycine) (Leu_2_Chg) with a one-carbon shorter side chain, showed a 32-fold decreased antibacterial activity compared to analogue **12**. This result suggests that the extra carbon is likely attributed to better flexibility and reach for interactions with the membrane. Analogue **14**, which contains the phenylalanine, also showed a decreased activity, suggesting the aromatic rings in position 2 of Novltex are not tolerated. This is evident as a similar trend was observed with analogue **15** (Leu_2_Tyr), where the addition of a hydroxy group to the aromatic ring further reduces the activity (>32 μg/mL). Likewise, analogue **13** (Leu_2_Trp), containing a bulky indole group, also showed poor activity (>32 μg/mL), indicating an increased size with polar atoms is not well tolerated on position 2 of Novltex. In contrast, analogue **16** (Leu_2_ d-*allo*-Ile) showed a 64-fold difference in MIC compared to analogue **12**. These results suggest that a flexible cyclohexyl ring side chain at position 2 of Novltex is required for potent antibacterial activity against MRSA.

We further evaluated analogue **12** against multidrug-resistant (MDR) strains of *S. aureus* (acquired from the NARSA collection) and MDR *E. faecium* (acquired from International Health Management Associates, IHMA, Europe Sarl), comparing its activity to various classes of clinically used antibiotics (Table 3).

**Table 3:**
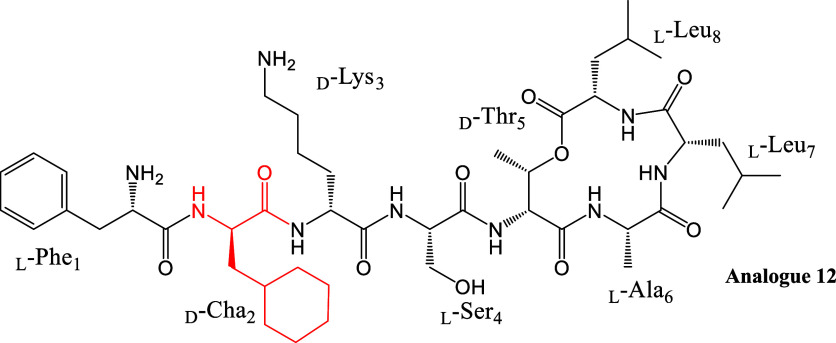

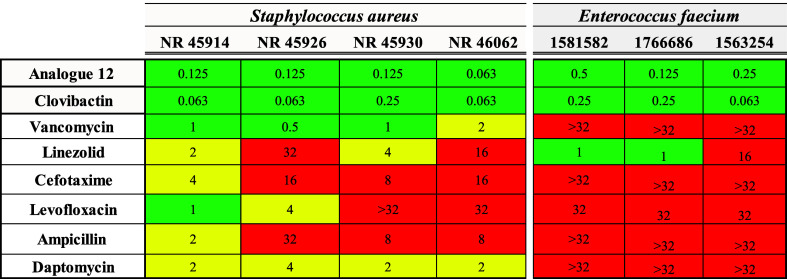
Antibacterial Activity of Novltex Analogue **12**, Clovibactin, and Clinically Used Antibiotics against Multidrug-Resistant (MDR) *S. aureus* and *E. faecium* (Clinical Isolates)^a^

a The colors represent the MIC activity profile: 0.0625–1 μg/mL (potent activity, green), 2–4 μg/mL (moderate activity, yellow), and 8–>32 μg/mL (poor activity, red).

Analogue **12** exhibited potent antibacterial activity (MIC = 0.063–0.5 μg/mL) against both groups of MDR clinical strains, with MIC values significantly lower or comparable to clinical antibiotics. *S. aureus* isolates displayed a range of susceptibility/resistance patterns for clinical antibiotics. Based on the MIC breakpoints, all the *S. aureus* isolates were susceptible to vancomycin and daptomycin, while 3 out of 4 isolates showed resistance to linezolid, cefotaxime, levofloxacin, and ampicillin.

Importantly, analogue **12** exhibited lower MIC values than the established susceptibility cutoff values for multiple antibiotics, confirming its superior antibacterial activity against MDR *S. aureus* compared to standard clinical treatments (Table S4, Section VII, Supporting Information). A similar effect was observed against MDR *E. faecium* strains. Compared to various clinically used antibiotics to which these strains exhibited resistance (vancomycin, cefotaxime, levofloxacin, ampicillin, and daptomycin; MIC range: 16 > 32 μg/mL), analogue **12** displayed potent antibacterial activity, with MIC values ranging from 0.125 to 0.5 μg/mL (Table 3). Notably, only two *E. faecium* clinical isolates remained susceptible to linezolid (MIC: 1 μg/mL).^19^

These findings suggest that analogue **12** exhibits a significantly higher potency than conventional antibiotics against MDR Gram-positive bacterial strains. Moreover, analogue **12** exhibited antibacterial activity comparable to that of Novo29/clovibactin against multidrug-resistant Gram-positive bacterial strains (Table 3).

### Time-Dependent Killing of Bacteria and Resistance Studies Using Novltex Analogue **12**

Early-stage time-kill kinetics for analogue **12** against MRSA ATCC 33591 were performed using vancomycin as a control, following the protocol described in Section VIII, Supporting Information. At 2.5 μg/mL (10× MIC, a desirable concentration at the site of infection), analogue **12** achieved significant bacterial killing within 6 h, whereas vancomycin, even at a higher concentration (5 μg/mL), required 24 h to produce a similar effect (Figure 3).

**Figure 3:**
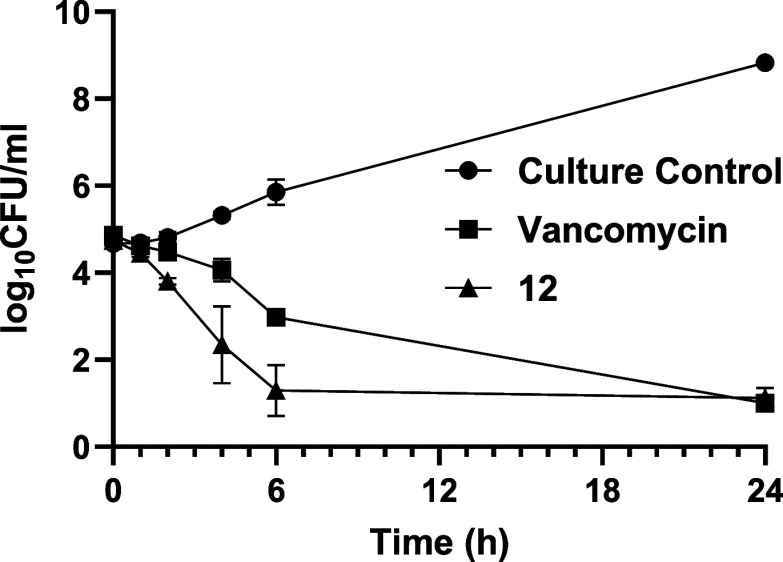
Time-kill kinetics of Novltex analogue **12** and clinical antibiotic (vancomycin) against MRSA 33591. The time-kill profile of analogue **12** at 2.5 μg/mL is comparable to that of vancomycin at an elevated concentration of 5.0 μg/mL. Results are the mean ± SD of two independent experiments performed in duplicate.

No resistant MRSA ATCC 33591 mutants emerged after exposure to 10× MIC Novltex analogue **12** on agar for 24 h at 37 °C.^11^

### Cytotoxicity and Hemolysis Assay of Analogue **12**

Analogue **12** was evaluated for cytotoxicity using human primary and cultured cell lines representing multiple cell types. The human primary dermal fibroblasts, hepatic cell line HepG2, and embryonic kidney cell line HEK293T treated with increasing concentrations of analogue **12** up to 50 μg/mL for 24 or 48 h showed no major cytotoxicity (Figure 4A–C, Section IX, Supporting Information), a concentration 100 times higher than the average MIC (0.5 μg/mL).

**Figure 4:**
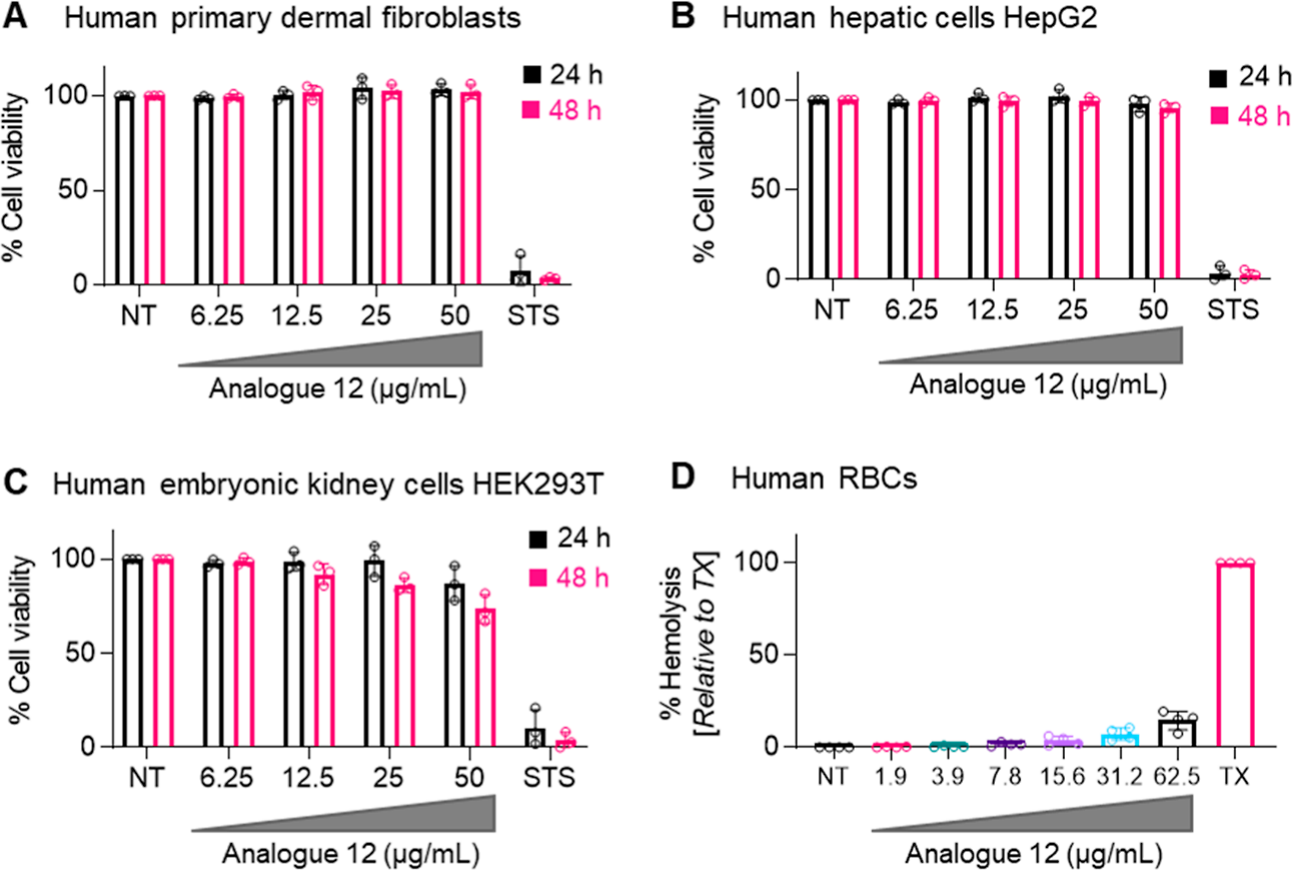
Cytotoxicity evaluation of analogue **12**. Human primary dermal fibroblasts (A), liver cell line HepG2 (B), and kidney cell line HEK293T (C) cells (5 × 10^3^ cells/well in 96-well plates) were treated with increasing concentrations of analogue **12** or 1 μM staurosporine (STS, used as a positive toxicity control) for 24 or 48 h, as indicated. Viability of cells was determined using an MTS-based assay. Results (% cell viability) are the mean ± SD of three independent experiments performed in triplicate. (D) Freshly collected human red blood cells (RBCs) were exposed to increasing concentrations of **12** or Triton X-100 (TX), as indicated, for 1 h. Hemolytic activity (% hemolysis) was determined by spectrophotometrically measuring the release of hemoglobin compared to that with TX (used as a positive control). Results are the mean ± SD of 4 independent experiments performed in duplicate using blood samples from four different anonymous healthy donors. NT, no treatment. Anonymized human blood samples were received from the Health Sciences Authority, Singapore, and used as per the institutional guidelines and approved by the Institutional Review Board (IRB-2023-1019) of Nanyang Technological University Singapore.

Additionally, an in vitro hemolysis assay against human erythrocytes (Figure 4D, Section X, Supporting Information) showed no detectable hemolysis at concentrations up to 31.2 μg/mL (>60× MIC), indicating good selectivity for bacterial cells. Even at an elevated concentration of 125× MIC (i.e., 62.5 μg/mL), analogue **12** induced low hemolysis (Figure 4D). These results demonstrate the potential suitability of analogue **12** for parenteral formulations.^20^

### Cytoplasmic Membrane Potential DisC_3_(5) Assay of Analogue **12**

To determine whether the bactericidal activity of analogue **12** is linked to bacterial membrane perturbations, we performed a DiSC_3_(5) assay, which utilizes a membrane-potential-sensitive dye to assess cytoplasmic membrane depolarization of MRSA.

As shown in Figure 5, a substantial increase in fluorescence intensity was observed with increasing concentrations of analogue **12**, indicating a disruption of the membrane potential. This effect became particularly pronounced at 4× MIC, confirming a significant membrane perturbation.

**Figure 5:**
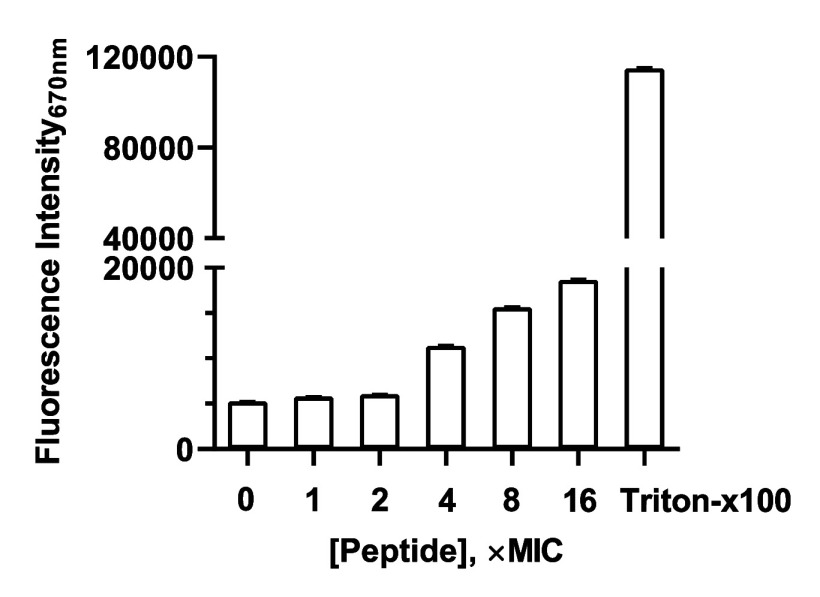
Change in fluorescence intensity of the membrane potential-sensitive probe, DiSC_3_(5), with increasing concentration of analogue **12** in addition to intact MRSA.

These results may suggest that analogue **12** impacts the cytoplasmic membrane potential of intact bacteria, in addition to targeting lipid II, contributing to its potent antibacterial activity.

### Lipid II Binding of Analogue **12** Using Spot-On Lawn Assays

To determine whether analogue **12** binds to lipid II, we performed in vitro lipid II-binding spot-on-lawn assays^21,22^ (Figure 6A and B).

**Figure 6:**
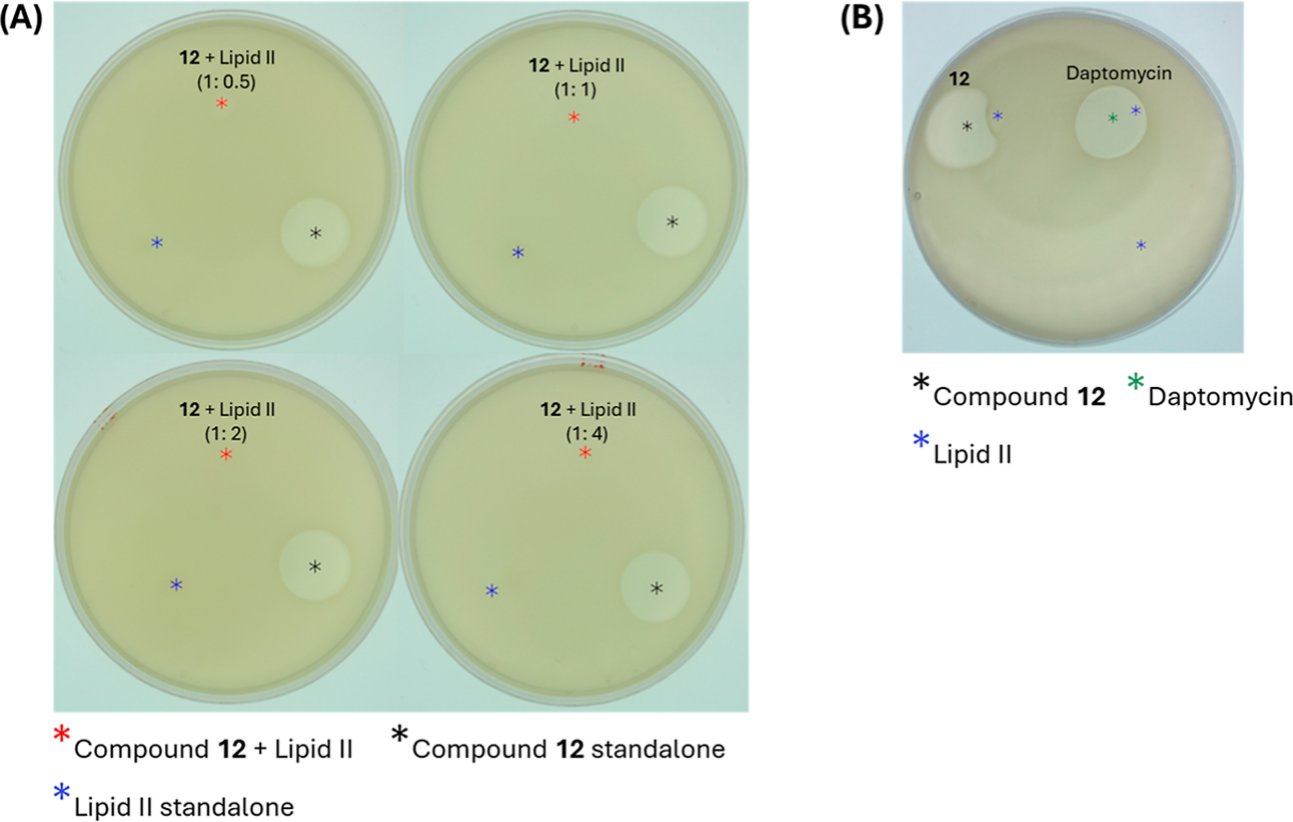
Spot-on-lawn assays to evaluate binding of Lipid II with analogue **12** on *S. aureus* ATCC 33591. (A) Analogue **12** was premixed with increasing ratios of lipid II (0.5 to 4 equiv) and spotted onto a seeded lawn of *S. aureus* 33591. Lipid II and analogue **12** were also spotted independently. The position of the analogue **12**–lipid II mixture is marked in red. (B) Daptomycin was used as a negative control. Asterisks denote the positions of analogue **12** (black), lipid II (blue), and daptomycin (green) additions.

### Lipid II Binding Determination by Inhibition Zone Reduction

Analogue **12** was premixed with increasing ratios of lipid II (0.5 to 4 equiv) for 10 min prior to spotting onto agar plates seeded with a lawn of *S. aureus* ATCC 33591 and incubated overnight. In the absence of lipid II, analogue **12** produced a clear and substantial zone of inhibition. At the lowest lipid II ratio (0.5), only a faint zone remained, and at a 1:1 ratio, antimicrobial activity was completely abolished. This progressive reduction in inhibition with an increase in lipid II concentration suggests sequestration of analogue **12**, confirming its ability to bind to lipid II (Figure 6A).

### Lipid II Binding Determination by Halo Distortion

To complement the inhibition zone reduction assay, we further assessed lipid II binding by observing halo distortion in a spot-on-lawn assay.

Upon the addition of lipid II, analogue **12** formed a noncircular spot, indicating a reduced inhibition zone in the presence of lipid II and disruption of the typical antibiotic-induced zone (halo). In contrast, daptomycin, which does not bind to lipid II, maintained a distinct circular inhibition zone, unaffected by the presence of lipid II (Figure 6B). These results suggest that analogue **12** binds to Lipid II.

## Conclusion

In conclusion, we have developed an efficient, high-yielding synthesis for Novltex, a novel class of antibiotics, utilizing low-cost, commercially available building blocks and achieving faster coupling times. This cost-effective methodology enabled the synthesis of a series of Novltex analogues, which were evaluated for their antibacterial activity against MRSA and multidrug-resistant (MDR) bacterial isolates.

Notably, Novltex analogue **12** emerged as a highly potent candidate, demonstrating superior antibacterial activity against MDR clinical isolates compared to that of existing classes of clinical antibiotics. Additionally, analogue **12** exhibited a favorable safety profile, reinforcing its potential for further development as a new treatment option against bacterial pathogens, including drug-resistant strains. Lipid II binding of analogue **12** confirms the preservation of the parent mechanism.

Furthermore, our study identified a clear correlation between the configurational patterns of amino acid compositions in Novltex analogues and their antibacterial efficacy, providing critical insights for future structural optimization.

This work lays a strong foundation for the development of next-generation Novltex analogues, which we are actively pursuing. These advancements hold significant promise for combating bacterial infections, addressing the growing AMR crisis, and ultimately improving and saving lives worldwide.

## Experimental Section

### Synthesis of Novltex Analogues

Novltex Analogue **4** was synthesized as described in (Scheme 1). (Step a) Commercially available 2-chlorotrityl chloride resin (manufacturer’s loading = 1.2 mmol/g, 200 mg resin) was swelled in DCM in a reactor. To this resin was added 4 eq. of Fmoc-Ala-OH/8 eq. of DIPEA in DCM, and the reactor was shaken for 3 h. The loading determined by UV absorption of the piperidine-dibenzofulvene adduct was calculated to be 0.6 mmol/g (220 mg resin, 0.132 mmol). Any unreacted resin was capped with MeOH/DIPEA:DCM = 1:2:7 by shaking for 1 h. (Step b) The Fmoc protecting group was deprotected using 20% piperidine in DMF by shaking for 3 min, followed by draining and shaking again with 20% piperidine in DMF for 10 min. AllocHN-d-Thr-OH was then coupled to the resin by adding 4 eq. of the AA, 4 eq. of HATU, and 8 eq. of DIPEA in DMF and shaking for 1 h at room temperature. (Step c) Esterification was performed using 10 eq. of Fmoc-Leu-OH, 10 eq. of DIC, and 5 mol % DMAP in DCM and shaking the reaction mixture for 1 h. This was followed by capping the unreacted alcohol using 10% Ac_2_O/DIPEA in DMF and shaking for 30 min, and Fmoc was removed using the protocol described earlier in step (b). (Step d) Fmoc-Leu-OH was coupled using 4 eq. of AA, 4 eq. of HATU, and 8 eq. of DIPEA in DMF and shaking for 1 h, followed by Fmoc deprotection using 20% piperidine in DMF as described earlier. (Step e) The N terminus of Leu was protected using 10 eq. of Trt-Cl and 15% Et_3_N in DCM and shaken for 1 h. The protection was verified by the ninhydrin color test. (Step f) The Alloc protecting group of d-Thr was removed using 0.2 eq. of [Pd­(PPh_3_)_4_]^0^ and 24 eq. of PhSiH_3_ in dry DCM under argon for 30 min. This procedure was repeated, increasing the time to 45 min, and the resin was washed thoroughly with DCM and DMF to remove any leftover Pd from the resin. (Step g) All amino acids were coupled using 4 eq. of amino acid and 4 eq. of DIC/Oxyma using a microwave peptide synthesizer. The coupling time was 10 min at 50 °C. Deprotection cycles were performed for 3 min at 50 °C followed by 10 min at RT. (Step h) The peptide was cleaved from the resin without cleaving off the protecting groups of the amino acid side chains using TFA/TIS:DCM = 2:5:93 and shaking for 1 h. (Step i) The solvent was evaporated, and the peptide was redissolved in DMF, to which 1 eq. of HATU and 10 eq. of DIPEA were added, and the reaction was stirred for 30 min to perform the cyclization. (Step j) The side-chain protecting groups were then cleaved off using TFA/TIS:H_2_O = 95:2.5:2.5 by stirring for 1 h. The peptide was precipitated using cold Et_2_O (−20 °C) and centrifuged at 7800 rpm to obtain a white solid. This solid was further purified using the equipment and methods described in Section II (Supporting Information), and pure fractions were pooled and freeze-dried to obtain a white solid (35 mg, 30% yield).

We synthesized all Novltex analogues using the method described above. The overall yields after HPLC purifications (monitored at 214 nm) were typically in the range of 25–30% (Scheme 1, Figure S1, Table S2, Sections III–V, Supporting Information).

Novltex analogue **4** was also characterized by NMR (Figures S35–S42, Table S3, Section VI, Supporting Information). The homogeneity of HPLC-purified fractions was analyzed by mass spectrometry. All of the Novltex analogues used were purified to >95% purity, as indicated by HPLC.

## Supplementary Material





## Notes

Structures of Novltex analogues, HPLC/LC–MS analysis results, NMR analysis results, antibacterial assay (MIC, time kill kinetics, resistance studies), cytotoxicity assay, hemolysis assay, cytoplasmic membrane potential assays, and lipid II binding assays (PDF)
Molecular formula strings of reported compounds (CSV)

## ‡‡

Department of Natural Sciences, Middlesex University, London, NW4 4BT, U.K

## Abbreviations

AA: amino acid
Ac_2_O: acetic anhydride
Alloc: allyloxycarbonyl
AMR: antimicrobial resistance
ATCC: American type cell culture
Boc: *tert*-butyloxycarbonyl
CFU: colony-forming unit
Cha: cyclohexyl alanine
Chg: cyclohexyl glycine
DCM: dichloromethane
DIC: diisopropylcarbodiimide
DIPEA/DIEA: diisopropylethylamine
DMAP: 4-dimethylaminopyridine;
DMF: *N*,*N*-dimethylformamide
Et_3_N: triethylamine
ESI: electrospray ionization
HATU: *N*-[(dimethylamino-1*H*-1,2,3-triazolo­[4,5-*b*]­pyridin-1-ylmethylene]-*N*-methylmethanaminium hexafluorophosphate *N*-oxide
HPLC: high-performance liquid chromatography
HRMS: high-resolution mass spectroscopy
MeOH: methanol
MIC: minimum inhibitory concentration
MRSA: methicillin-resistant *Staphylococcus aureus*
*E. faecium*: *Enterococcus faecium*
NMR: nuclear magnetic resonance
PBS: phosphate-buffered saline
[Pd­(PPh_3_)_4_]^0^: tetrakis­(triphenylphosphine)­palladium­(0)
PG: protecting group
PhSiH_3_: phenylsilane
RBC: red blood cell
*S. aureus* (SA): *Staphylococcus aureus*
STS: staurosporine
TFA: trifluoroacetic acid
TIS: triisopropylsilane
Trt: trityl
TX: triton X-100.
