# Supplementary material for: Novltex: A New Class of Antibiotics with Potent Activity against Multidrug-Resistant Bacterial Pathogens—Design, Synthesis, and Biological Evaluation
Source: J Med Chem. 2025 Sep 16;68(18):19143–52. doi: 10.1021/acs.jmedchem.5c01193 (PMC12481569; doi:10.1021/acs.jmedchem.5c01193)
Supplement: Supplementary file 1 [file jm5c01193_si_001.pdf]

Supporting Information for

Novltex: A New Class of Antibiotics with Potent Activity against Multidrug-Resistant Bacterial Pathogens—Design, Synthesis, and Biological Evaluation

Esra Malkawi[†,‡,¶,#], Anish Parmar[†,‡,#], Sanjit Das[†,‡,#], Enas Newire[†,Ø,#], Charlotte M. Jones[†,‡], Kate A. Morrison[‡], Milandip Karak[ð], Frédéric Blanc[‡,§,Ÿ], Nicholas Harper[†], Rajamani Lakshminarayanan[Ž,¢, ⊥], Zhi Sheng Poh[ǁ], Navin K. Verma[ǁ], Jennifer Unsworth[†], Dallas E. Hughes[þ], Losee Lucy Ling[þ], Stephen A. Cochrane[ð], William Hope[†] and Ishwar Singh[*,†,‡]

[†] Antimicrobial Pharmacodynamics and Therapeutics, Department of Pharmacology and Therapeutics, William Henry Duncan Building, University of Liverpool, Liverpool L7 8TX, U.K.

[‡] Department of Chemistry, University of Liverpool, Liverpool L69 7ZD, U.K.

[¶] College of Pharmacy, Amman Arab University, Amman 11953, Jordan

[ð] School of Chemistry and Chemical Engineering, Queen's University Belfast, David Keir Building, Stranmillis Road, Belfast BT9 5AG, U.K.

[§] Leverhulme Research Centre for Functional Materials Design, Materials Innovation Factory, University of Liverpool, Liverpool L7 3NY, U.K.

[Ÿ] Stephenson Institute for Renewable Energy, University of Liverpool, Liverpool L69 7ZF, U.K.

[Ž] Singapore Eye Research Institute, The Academia, Discovery Tower Level 6, 20 College Road, Singapore 169857, Singapore.

[¢]Department of Pharmacy & Pharmaceutical Sciences, National University of Singapore, 117543, Singapore.

[⊥] Ophthalmology and Visual Sciences Academic Clinical Program, Duke-NUS Graduate Medical School, Singapore 169857, Singapore.

[ǁ] Lee Kong Chian School of Medicine, Nanyang Technological University Singapore, 11 Mandalay Road, Singapore 308232, Singapore.

[þ] NovoBiotic Pharmaceuticals, Cambridge, Massachusetts 02138, United States.

[#] Contributed equally; Present addresses: [Ø] Department of Natural Sciences, Middlesex University, London, NW4 4BT, U.K.

*Corresponding author: Ishwar Singh, Email: i.singh@liverpool.ac.uk

Table of Contents

I. Materials 3

II. Equipment used for the analysis and purification of compounds 3

III. Synthetic scheme of Novltex analogues 5

IV. Structures 7

V. HPLC/LC-MS analysis 8

VI. NMR data 25

VII. MIC testing 31

VIII. Time dependent killing of bacteria and resistance studies by analogue 12 31

IX. Cytotoxicity Assay 31

X. Hemolysis Assay 32

XI. Cytoplasmic membrane potential $DiSC_3(5)$ assay 32

XII. Lipid II binding determination of analogue 12 33

XIII. References 33

I. Materials

All L amino acids, Fmoc-D-Leu-OH, Fmoc-D-Lys(Boc)-OH, Fmoc-D-Ser(tBu)-OH, Boc-D-phenylalanine, 1-[Bis(dimethylamino)methylene]-1H-1,2,3-triazolo[4,5-b]pyridinium3-oxidhexafluorophosphate (HATU), Phenylsilane ($PhSiH_3$), Diisopropylethylamine (DIPEA), Tritylchloride 4-(Dimethylamino)pyridine(DMAP), Tetrakis(triphenylphosphine)palladium(0) [$Pd(PPh_3)$], Ethyl cyano(hydroxyimino)acetate (Oxyma Pure), Diisopropylcarbodiimide (DIC) and Triisopropylsilane (TIS) were purchased from Fluorochem, UK. Dimethylformamide (DMF) peptide synthesis grade was purchased from Rathburn chemicals. Triethylamine, Diethyl ether (Et_2O), Dimethylsulfoxide (DMSO), Dichloromethane (DCM), Formic acid 98-100% purity, Water (HPLC grade) and Acetonitrile (HPLC grade) were purchased from Fisher Scientific. 2-Chlorotritylchloride resin (manufacturer's loading: 1.20 mmol/g) was purchased from Iris Biotech GmbH. All chemicals were used without further purification. Gram-positive lipid II was synthesised chemically according to previously published methods[1]. Natural Clovibactin was isolated by previously reported methods[2].

II. Equipment used for the analysis and purification of compounds

All peptides were analysed on a Thermo Scientific Dionex Ultimate 3000 RP-HPLC equipped with a Phenomenex Gemini NX C18 110 Å (150 x 4.6 mm) column using the following buffer systems: A: 0.1% HCOOH in milliQ water. B: ACN using a flow rate of 1 mL/min. Detection was performed at 214 nm. The column was flushed with 95% A for 5 min prior to an injection and was flushed for 5 min with 95% B and 5% A after the run was finished.

Peptides were analysed using the following gradient: 95% A for 2 min. 5-95% B in 25 min. 95% B for 5 min. 5% A for 4 min.

Peptides were purified using the same gradient as mentioned above, on a Biotage® Isolera one flash purification system with a flow rate of 25 mL/min and monitored at 214 nm.

LC-MS data were collected on a Thermo Scientific Dionex Ultimate 3000 RP-UPLC instrument with a Phenomenex Kinetex C18 100Å column (50 x 2.1 mm, 2.6 µm at 30 °C) connected to a Thermo Scientific™ ISQ™ EC Single Quadrupole Mass Spectrometer with a flow rate of 0.6 mL/min with the following solvent systems: (A): 0.1% HCOOH in H_2O and (B) ACN. Detection was performed at 214 nm. The column was flushed with 95% A for 2 min, then a gradient from 5% A to 95% B over 6 min was used, followed by 2 min of flushing with 95% B.

NMR spectra of Novltex analogue **4** were collected at 20 °C on a Bruker Avance III HD 500 spectrometer equipped with a liquid nitrogen-cooled CryoProbe Prodigy™ and referenced against the residual solvent peaks of DMSO-d_6 at 2.50 ppm for ^1H and 39.53 ppm for ^{13}C. A 3 mM solution of Novltex analogue **4** in DMSO-d_6 was prepared by dissolving 1.3 mg of the peptide in 0.5 mL of solvent.

Spectral assignments of Novltex **4** were obtained from an exhaustive NMR data set combining ^{1}H, ^{13}C{^{1}H}, ^{1}H ^{1}H COSY (Fig. S36), ^{1}H ^{1}H NOESY (Fig. S37), ^{1}H ^{1}H TOCSY (Fig. S38), ^{1}H ^{13}C HSQC (Fig. S40) and ^{1}H ^{13}C HMBC (Fig. S41) and are given in Table S3. Homonuclear, HSQC and HMBC correlations were collected with 8, 64 and 8 scans, respectively, 2048 t_2 points and 512 t_1 increments. A significant number of scans (8672) were acquired for the ^{13}C{^{1}H} spectrum. Mixing and spinlock mixing times of 200 and 150 ms were used in the NOESY and TOCSY, respectively. COSY, NOESY and HSQC were acquired with non-uniform sampling with 25% sparsity of data points generated randomly and processed with compressed sensing as implemented in Topspin[3]. Spectra were processed by phasing both dimensions followed by automatic base line correction, and all data were zero-filled to four times the number of points in the time domains. ^{1}H and ^{13}C time domains were apodised with Qsine and exponential decay weighting functions, respectively, and forward linear prediction in indirect dimensions were used.

III. Synthetic scheme of Novltex analogues

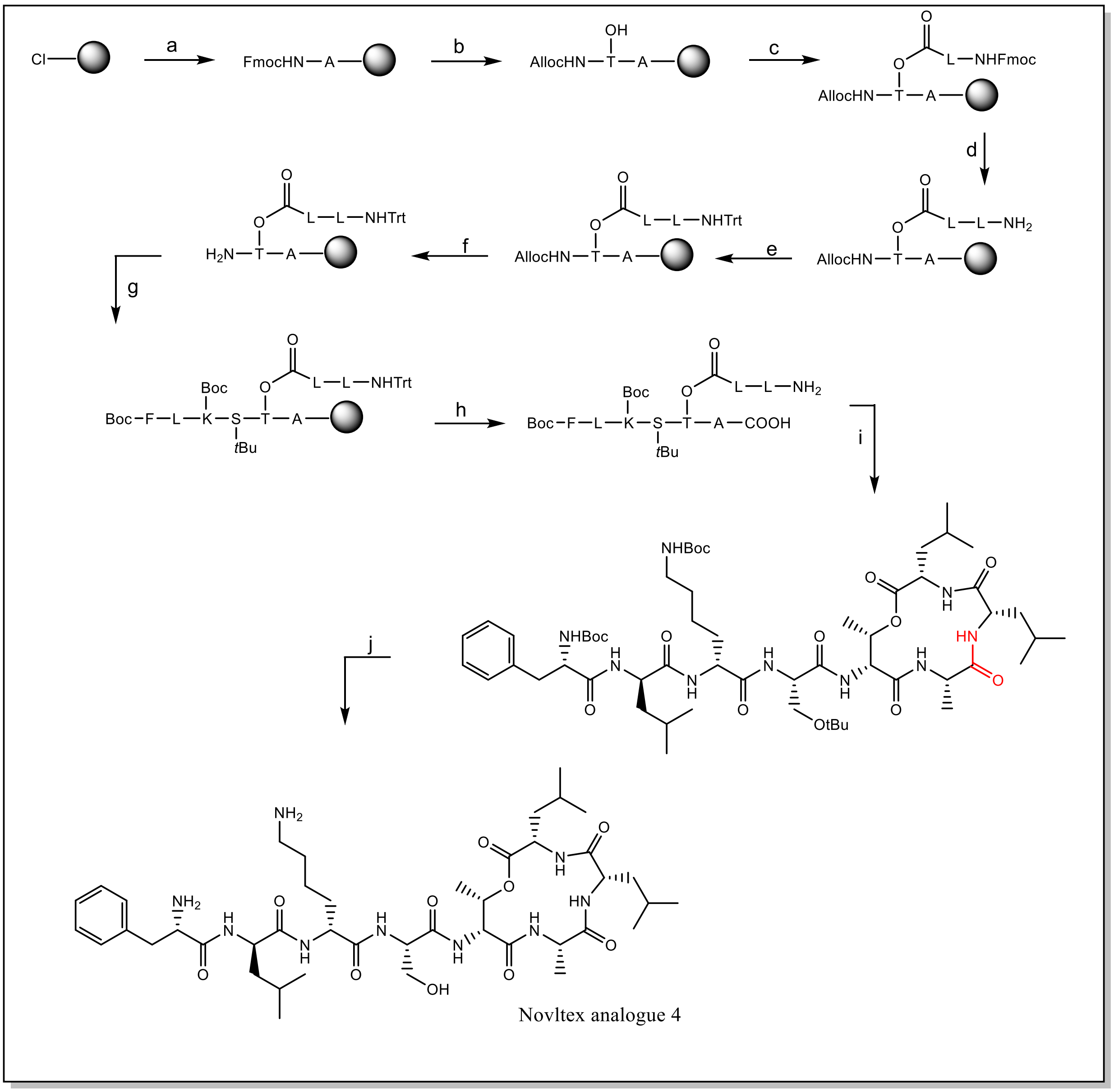

Fig. S1: Synthesis of Novltex analogue **4**.

(step a) Commercially available 2-Chlorotrityl chloride resin (manufacturer's loading = 1.2 mmol/g, 200 mg resin) was swelled in DCM in a reactor. To this resin was added 4 eq. Fmoc-Ala-OH/8 eq. DIPEA in DCM and the reactor was shaken for 3 h. The loading determined by UV absorption of the piperidine-dibenzofulvene adduct was calculated to be 0.6 mmol/g, (220 mg resin, 0.132 mmol). Any unreacted resin was capped with MeOH:DIPEA:DCM = 1:2:7 by shaking for 1h. (step b) The Fmoc protecting group was deprotected using 20% piperdine in DMF by shaking for 3 min, followed by draining and shaking again with 20% piperidine in DMF for 10 min. AllocHN-D-Thr-OH was then coupled to the resin by adding 4 eq. of the AA, 4 eq. HATU and 8 eq. DIPEA in DMF and shaking for 1 h at room temperature. (step c) Esterification was performed using 10 eq. of Fmoc-Leu-OH, 10 eq.

DIC and 5 mol% DMAP in DCM and shaking the reaction for 1 h. This was followed by capping the unreacted alcohol using 10% Ac_2O/DIPEA in DMF shaking for 30 min and Fmoc was removed using protocol described earlier in step (b). (step d) Fmoc-Leu-OH was coupled using 4 eq. of AA, 4 eq. HATU and 8 eq. DIPEA in DMF and shaking for 1h followed by Fmoc deprotection using 20% piperidine in DMF as described earlier. (step e) The N terminus of Leu was protected using 10 eq. Trt-Cl and 15% Et_3N in DCM and shaking for 1h. The protection was verified by the Ninhydrin colour test. (step f) The Alloc protecting group of D-Thr was removed using 0.2 eq. $[Pd(PPh_3)_4]^0$ and 24 eq. $PhSiH_3$ in dry DCM under argon for 30 min. This procedure was repeated increasing the time to 45 min and the resin was washed thoroughly with DCM and DMF to remove any leftover Pd on resin. (step g) All amino acids were coupled using 4 eq. Amino Acid, 4 eq. DIC/Oxyma using a microwave peptide synthesiser. Coupling time was 10 min @ 50°C. Deprotection cycles were performed 3 min @ 50°C followed by 10 min @ RT. (step h) The peptide was cleaved from the resin without cleaving off the protecting groups of the amino acid side chains using TFA:TIS:DCM = 2:5:93 and shaking for 1 h. (step i) The solvent was evaporated and the peptide was redissolved in DMF to which 1 eq. HATU and 10 eq. DIPEA were added and the reaction was stirred for 30 min to perform the cyclisation. (step j) The side-chain protecting groups were then cleaved off using TFA:TIS:H_2O = 95:2.5:2.5 by stirring for 1h. The peptide was precipitated using cold Et_2O (-20°C) and centrifuging at 7800 rpm to obtain a white solid. This solid was further purified using the equipment and methods described in section II and pure fractions were pooled and freeze dried to obtain a white solid 35 mg, 30% yield.

IV. Structures

Analogue 1

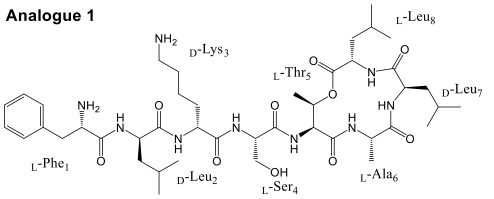

Analogue 2

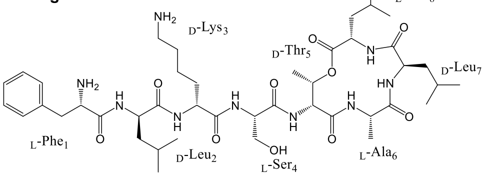

Analogue 3

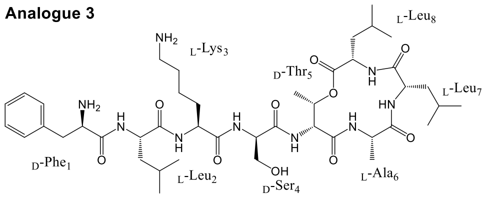

Analogue 4

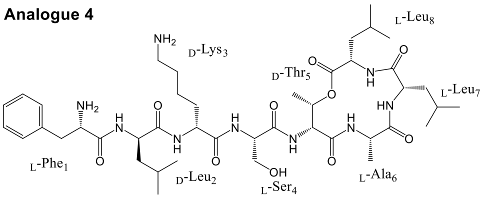

Analogue 5

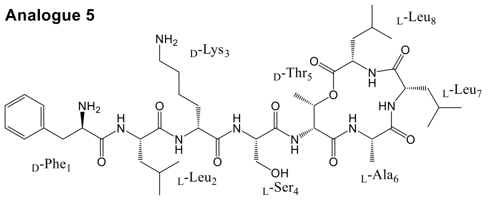

Analogue 6

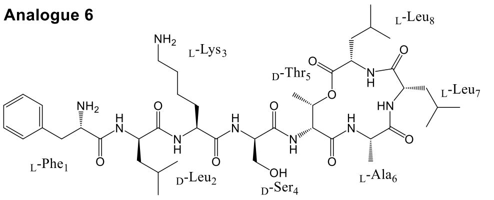

Analogue 7

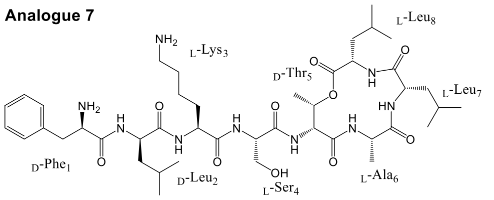

Analogue 8

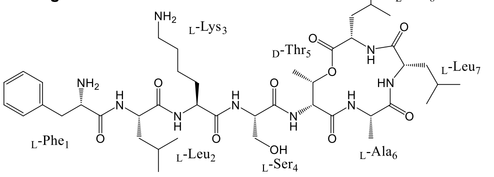

Analogue 9

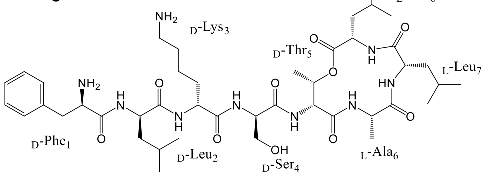

Analogue 10

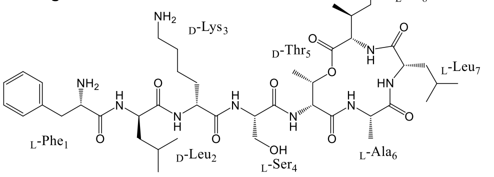

Analogue 11

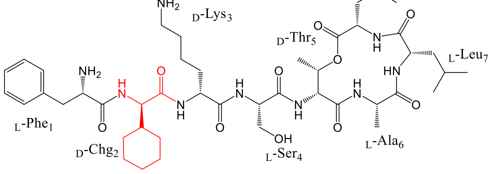

Analogue 12

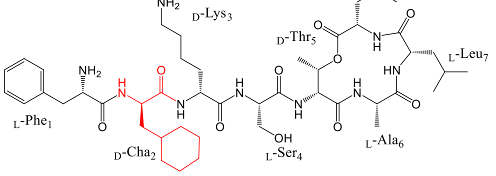

Analogue 13

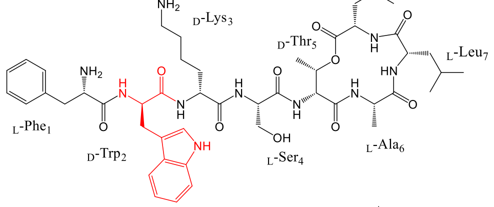

Analogue 14

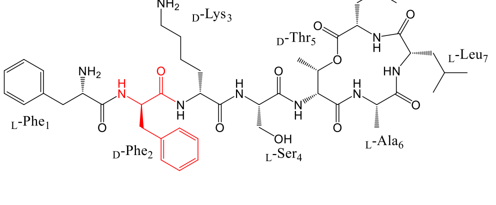

Analogue 15

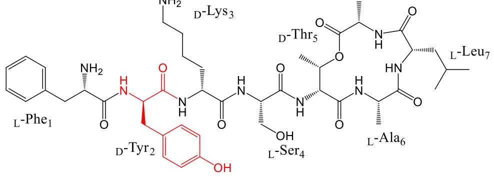

Analogue 16

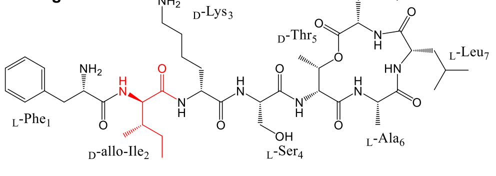

Fig. S2: Structures of Novltex analogues **1-16.**

V. HPLC/LC-MS analysis

Analogue No.	Code	*Amino acid Configuration*							
	**TAIL-RING**	**Phe1**	**Leu2**	**Lys3**	**Ser4**	**Thr5**	**Ala6**	**Leu7**	**Leu8**
1	LDDL-LLDL	L	D	D	L	L	L	D	L
2	LDDL-DLDL	L	D	D	L	D	L	D	L
3	DLLD-DLLL	D	L	L	D	D	L	L	L
4	LDDL-DLLL	L	D	D	L	D	L	L	L
5	DLDL-DLLL	D	L	D	L	D	L	L	L
6	LDLD-DLLL	L	D	L	D	D	L	L	L
7	DDLL-DLLL	D	D	L	L	D	L	L	L
8	LLLL-DLLL	L	L	L	L	D	L	L	L
9	DDDD-DLLL	D	D	D	D	D	L	L	L
10†	LDDL-DLLL	L	D	D	L	D	L	L†	L

Table. S1: Amino acid configuration of Novltex analogues **1-10**

Analogue No.	Exact Mass	Mass found [M + H+]
1	873.53	874.9
2	873.53	874.9
3	873.53	874.9
4	873.53	874.5
5	873.53	874.9
6	873.53	875.1
7	873.53	875.2
8	873.53	874.6
9	873.53	874.9
10†	873.53	874.7
11	899.54	900.7
12	913.56	914.8
13	946.52	947.8
14	907.51	908.7
15	923.51	925.5
16	873.53	874.8

Table. S2: Mass analysis for compounds **1-16**. †Indicates ***Leu8*** replaced by ***Ile8***. Compounds **11-16** have the same configuration as analogue **4**. Overall yields in the range of 25-30%.

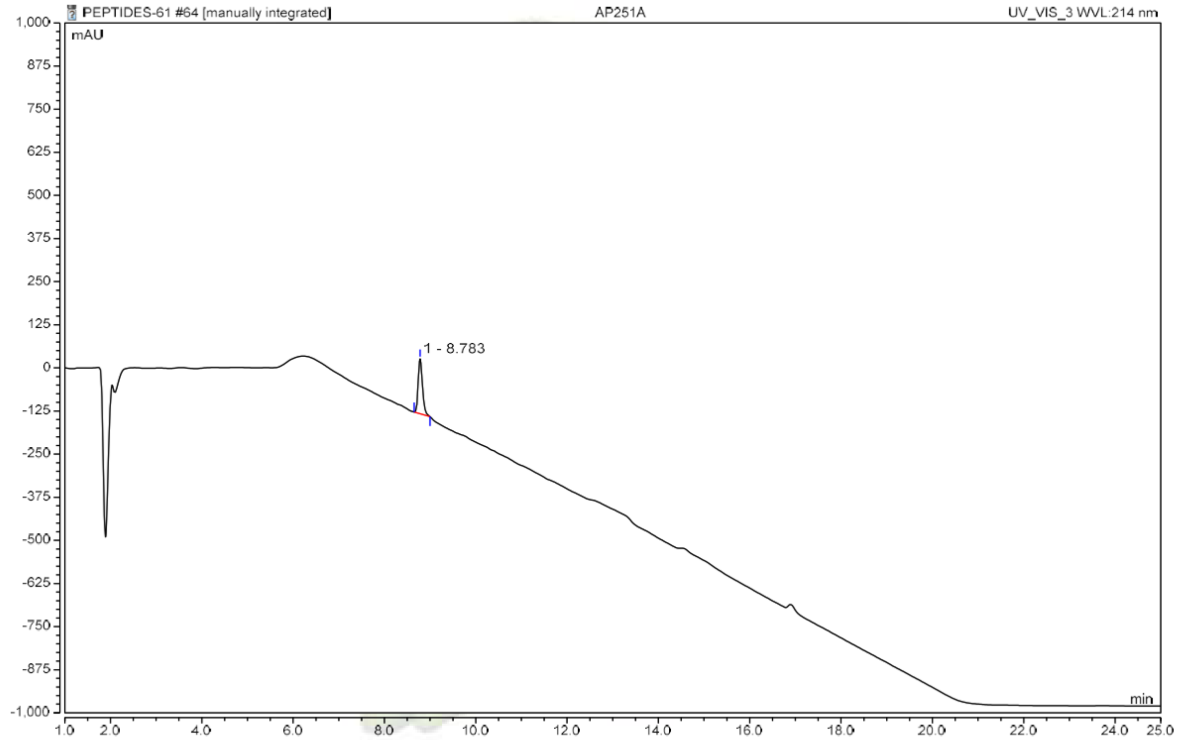

Fig. S3: HPLC trace of HPLC purified Novltex analogue **1** (gradient: 5–95% ACN in 25 min using A: 0.1% HCOOH in water, B: ACN).

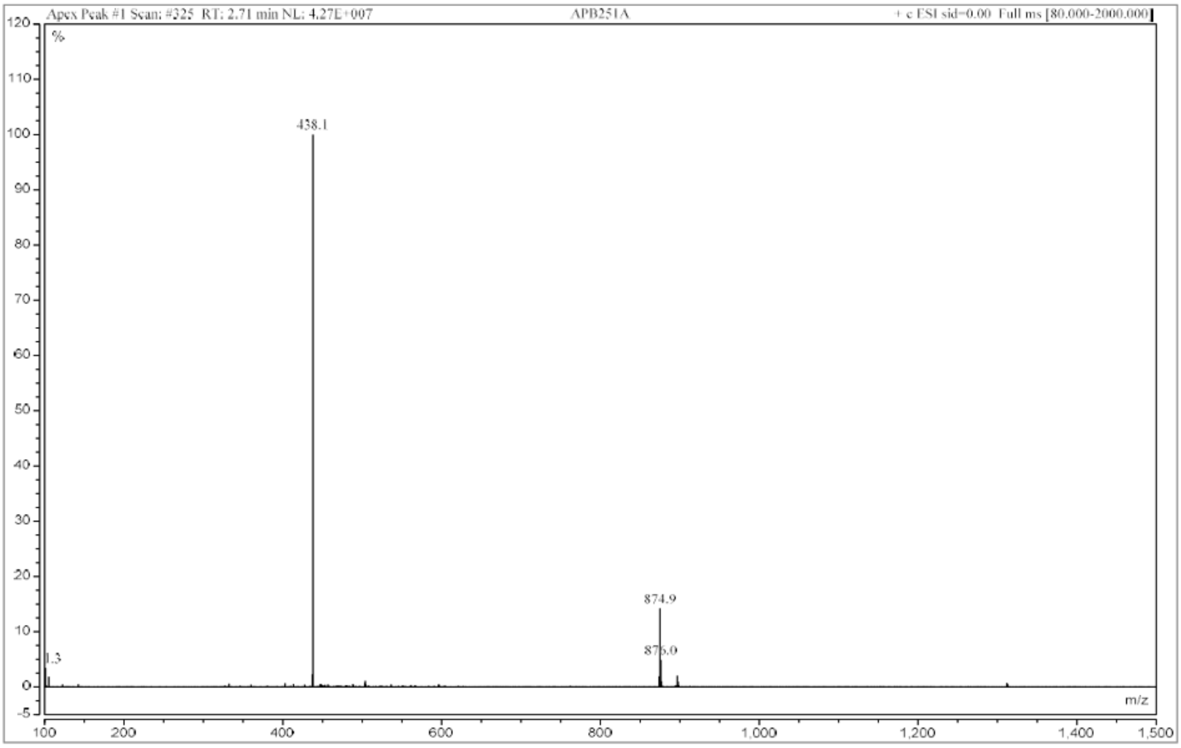

Fig. S4: MS spectra of HPLC purified Novltex analogue **1**. Exact Mass calcd. for $C_{43}H_{71}N_9O_{10}$ = 873.53, found $M+H^+$ = 874.9 and $M/2 + H^+$ = 438.1

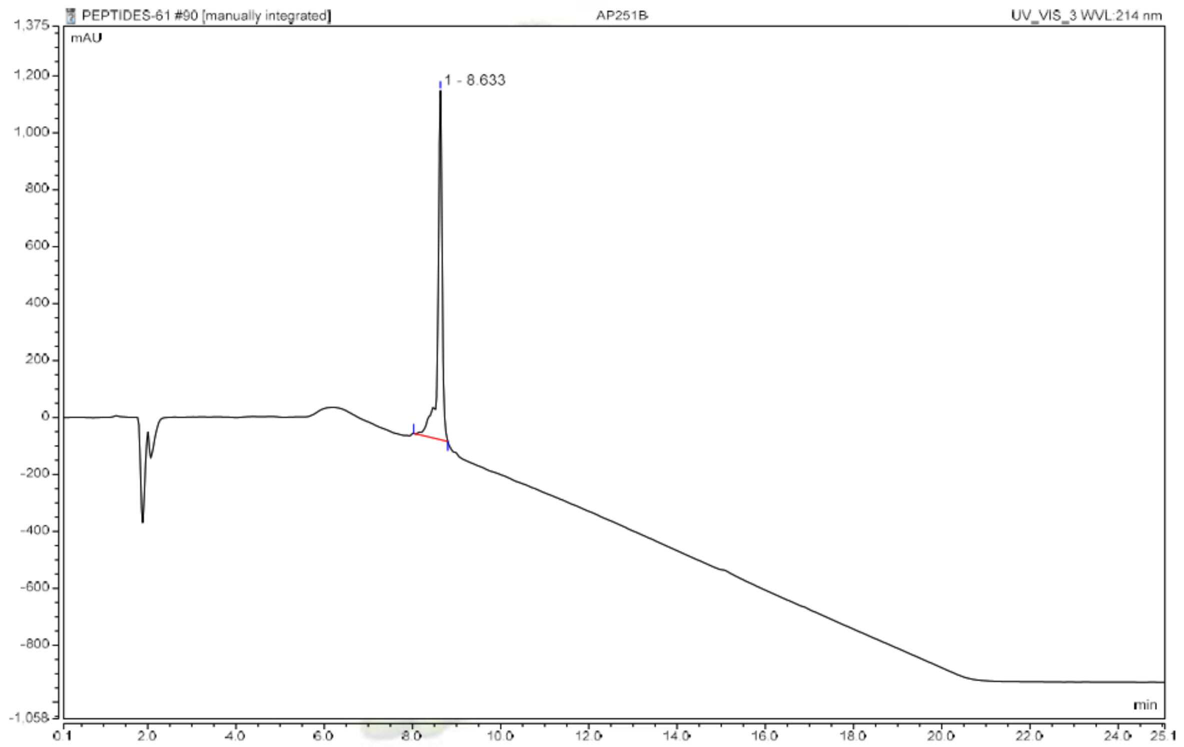

Fig. S5: HPLC trace of HPLC purified Novltex analogue **2** (gradient: 5–95% ACN in 25 min using A: 0.1% HCOOH in water, B: ACN).

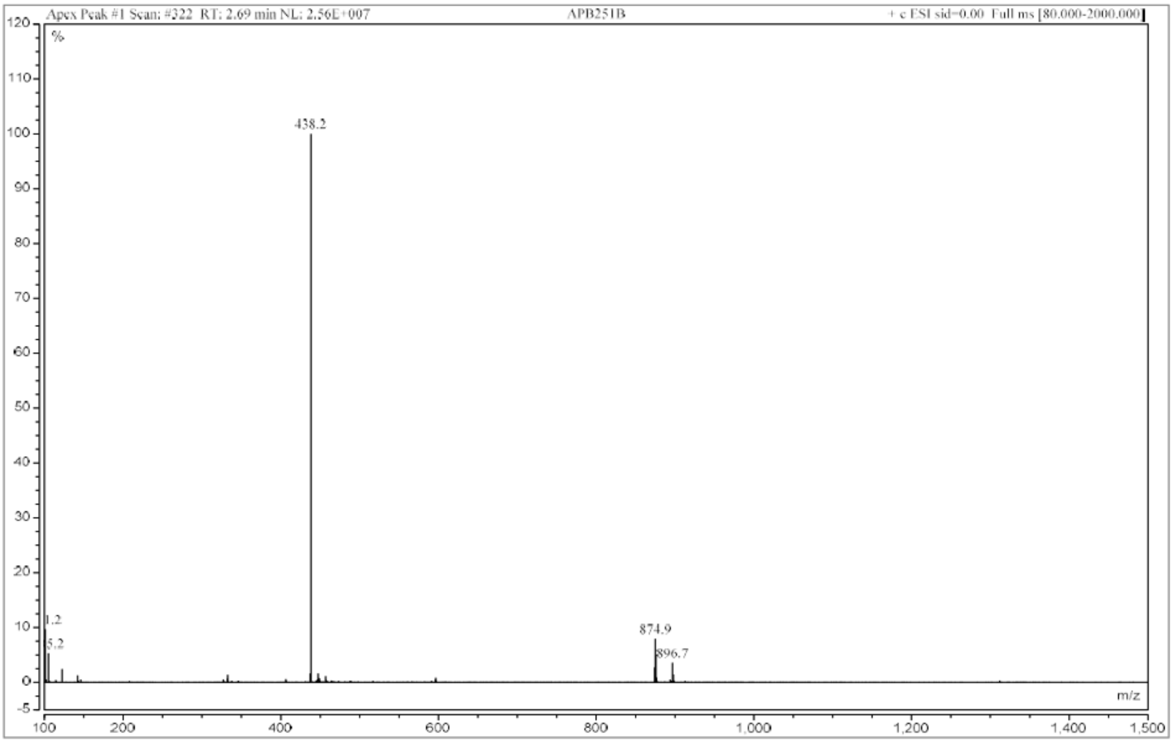

Fig. S6: MS spectra of HPLC purified Novltex analogue **2**. Exact Mass calcd. for $C_{43}H_{71}N_9O_{10}$ = 873.53, found $M+H^+$ = 874.9 and $M/2 + H^+$ = 438.2

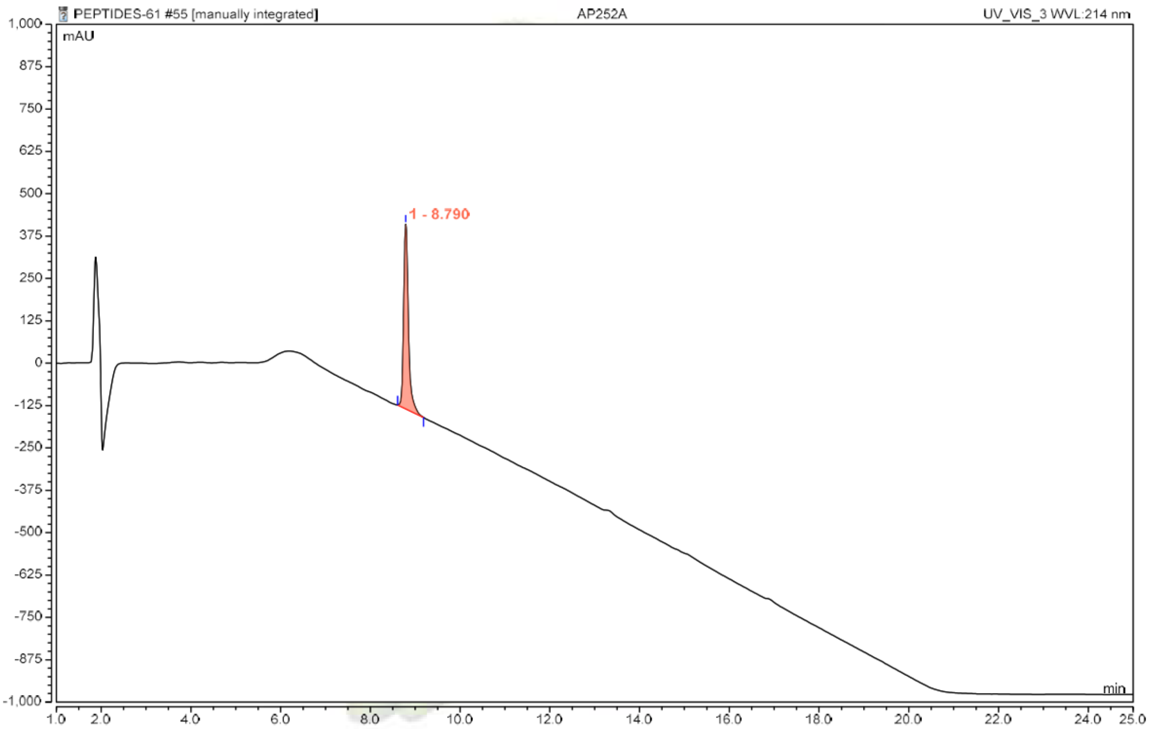

Fig. S7: HPLC trace of HPLC purified Novltex analogue **3** (gradient: 5–95% ACN in 25 min using A: 0.1% HCOOH in water, B: ACN).

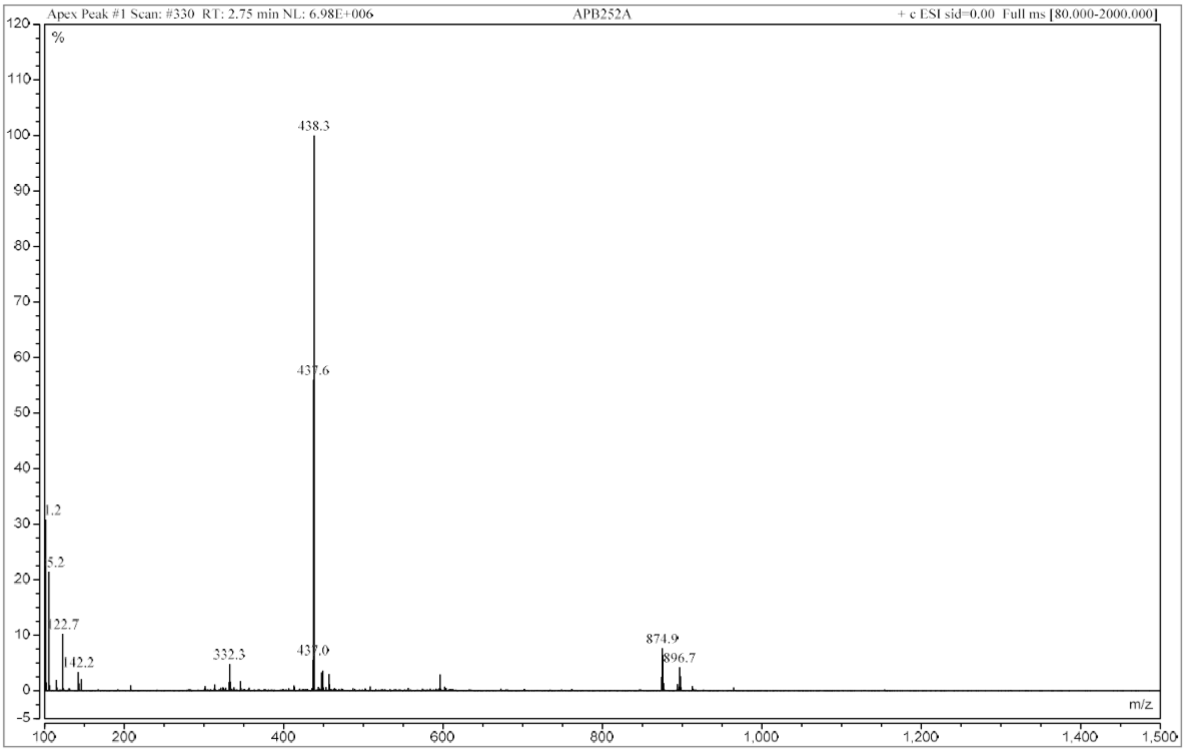

Fig. S8: MS spectra of HPLC purified Novltex analogue **3**. Exact Mass calcd. for $C_{43}H_{71}N_9O_{10}$ = 873.53, found $M+H^+$ = 874.9 and $M/2 + H^+$ = 438.3

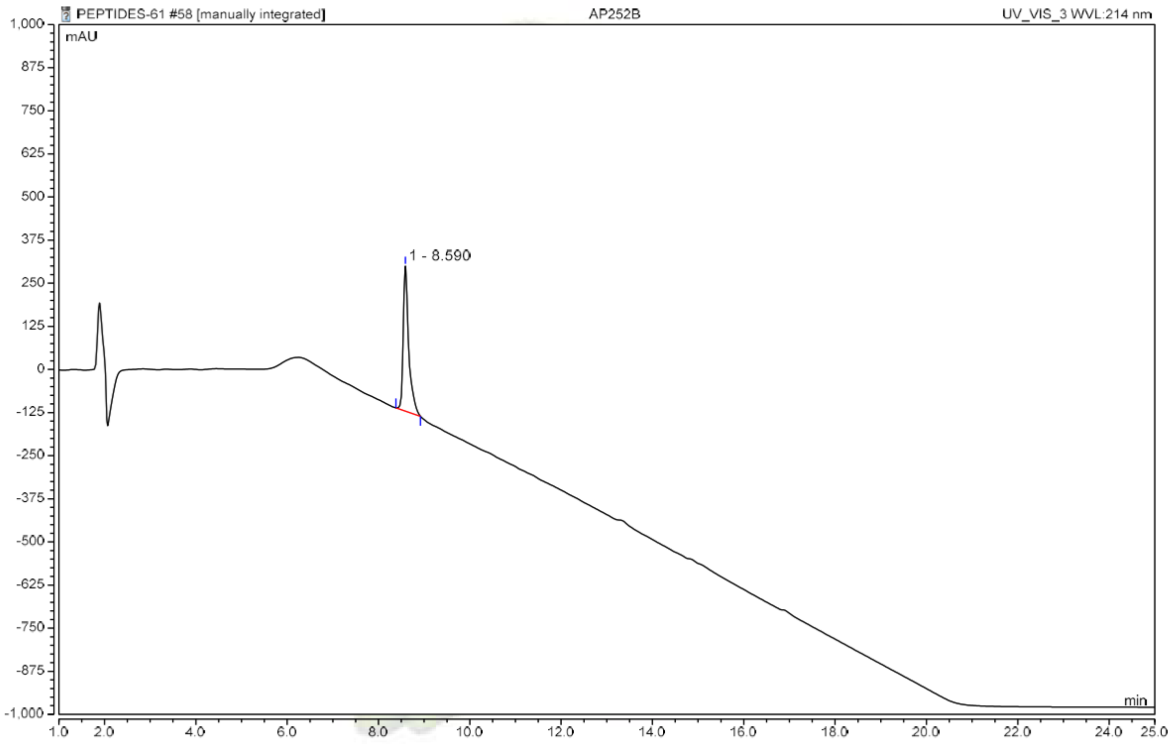

Fig. S9: HPLC trace of HPLC purified Novltex analogue **4** (gradient: 5–95% ACN in 25 min using A: 0.1% HCOOH in water, B: ACN).

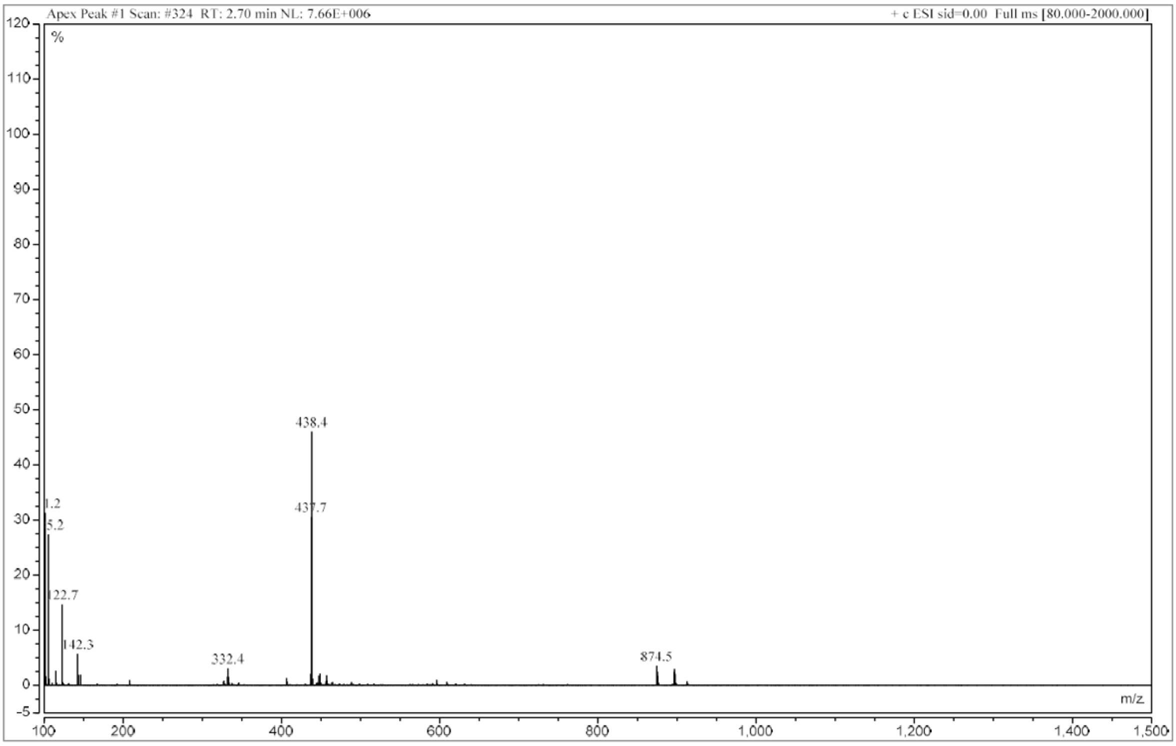

Fig. S10: MS spectra of HPLC purified Novltex analogue **4**. Exact Mass calcd. for $C_{43}H_{71}N_9O_{10}$ = 873.53, found $M+H^+$ = 874.5 and $M/2 + H^+$ = 438.4

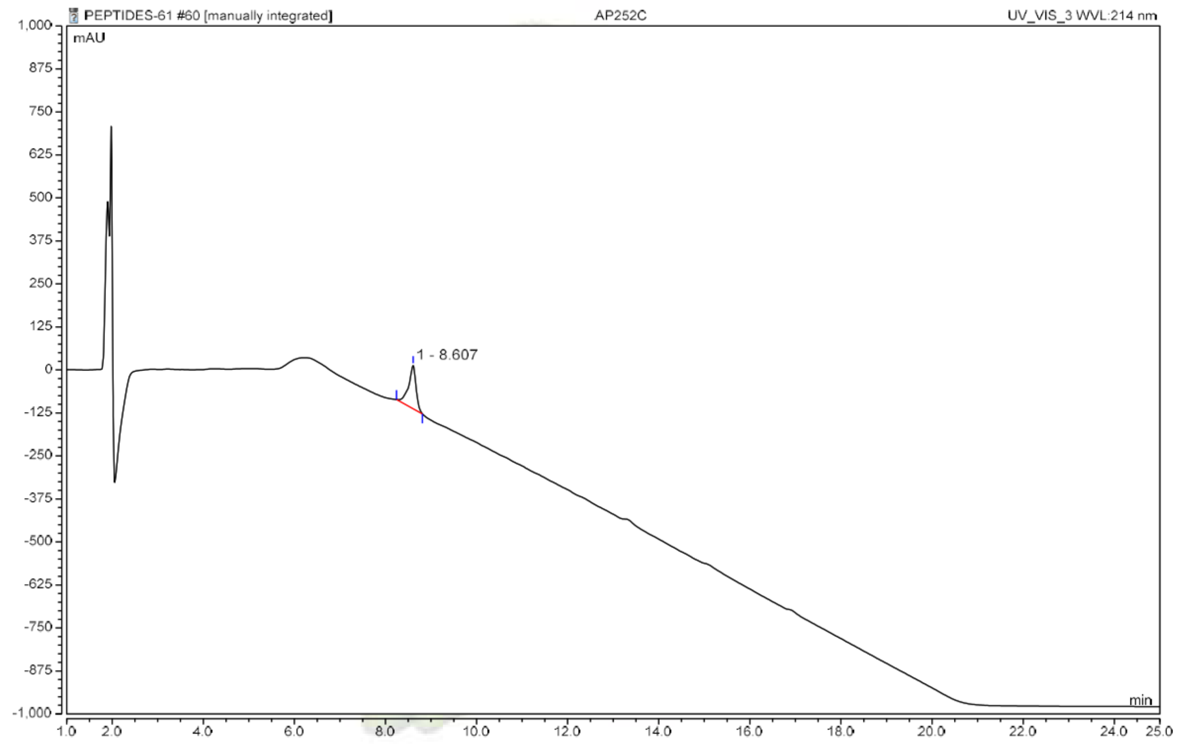

Fig. S11: HPLC trace of HPLC purified Novltex analogue 5 (gradient: 5–95% ACN in 25 min using A: 0.1% HCOOH in water, B: ACN).

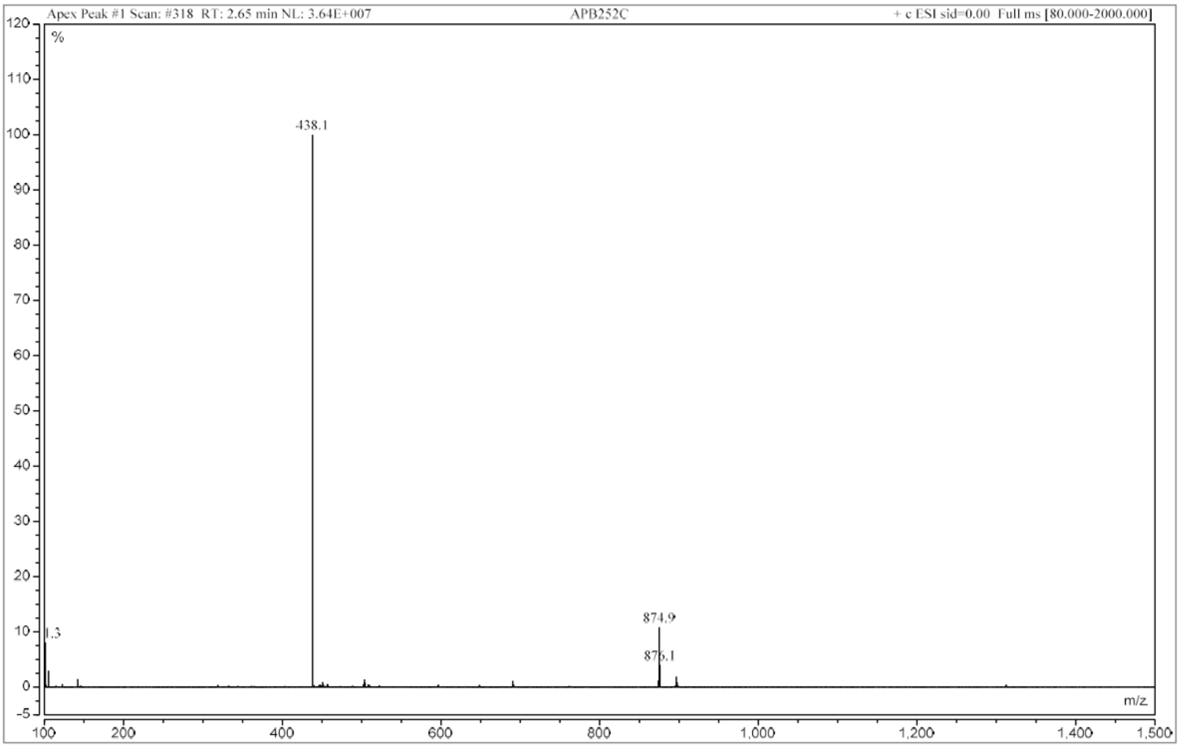

Fig. S12: MS spectra of HPLC purified Novltex analogue **5**. Exact Mass calcd. for $C_{43}H_{71}N_9O_{10}$ = 873.53, found M+H$^+$ = 874.9 and M/2 + H$^+$ = 438.1

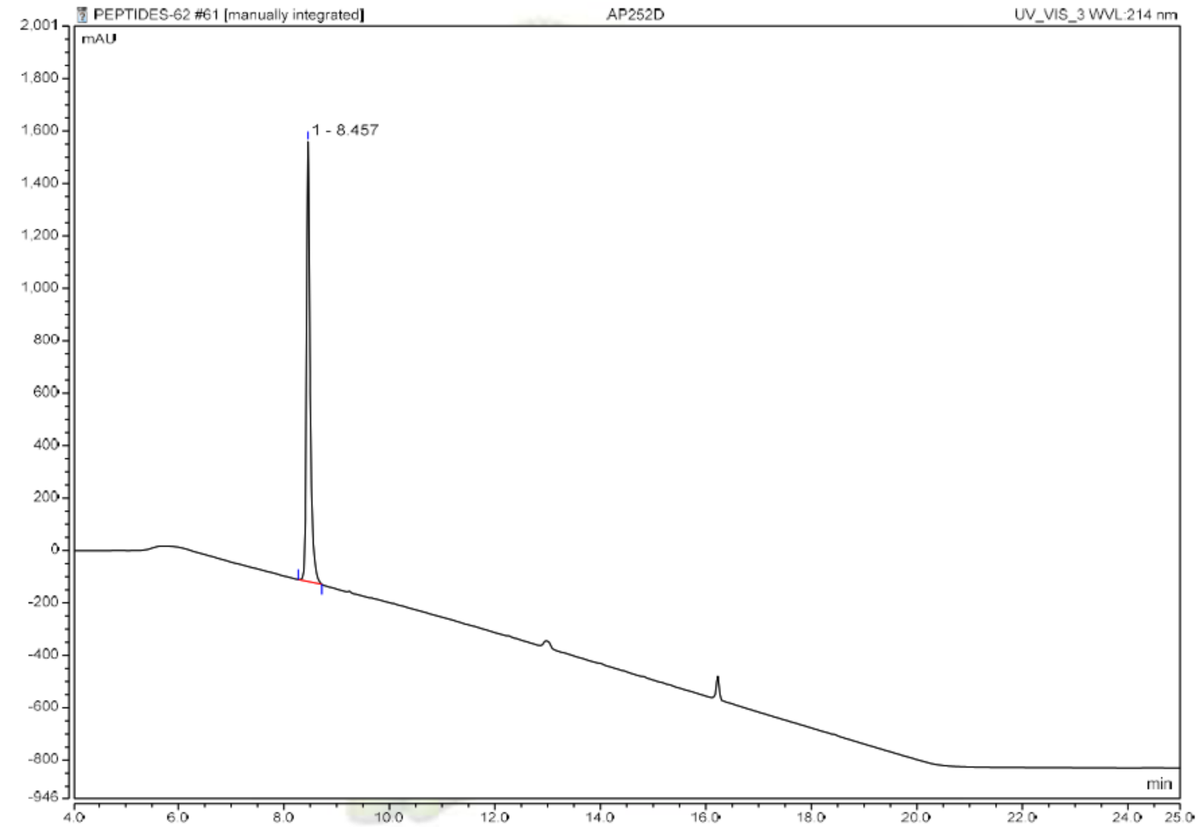

Fig. S13: HPLC trace of HPLC purified Novltex analogue 6 (gradient: 5–95% ACN in 25 min using A: 0.1% HCOOH in water, B: ACN).

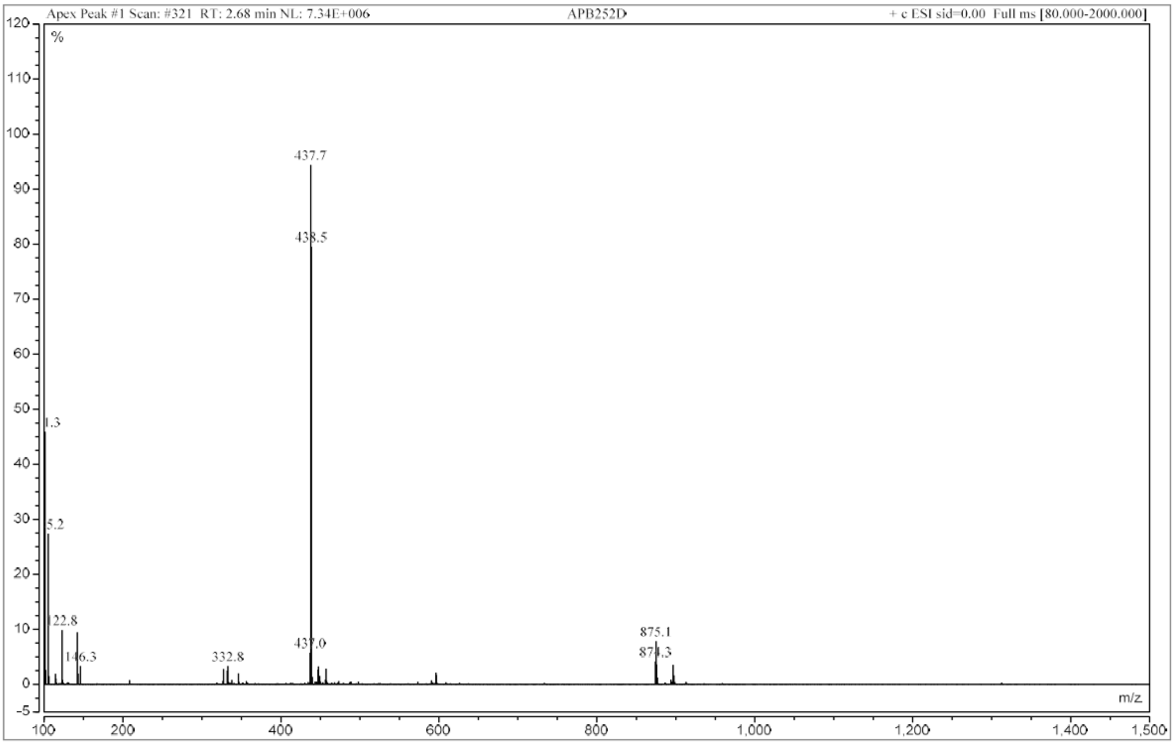

Fig. S14: MS spectra of HPLC purified Novltex analogue 6. Exact Mass calcd. for $C_{43}H_{71}N_9O_{10}$ = 873.53, found $M+H^+$ = 875.1 and $M/2 + H^+$ = 437.7

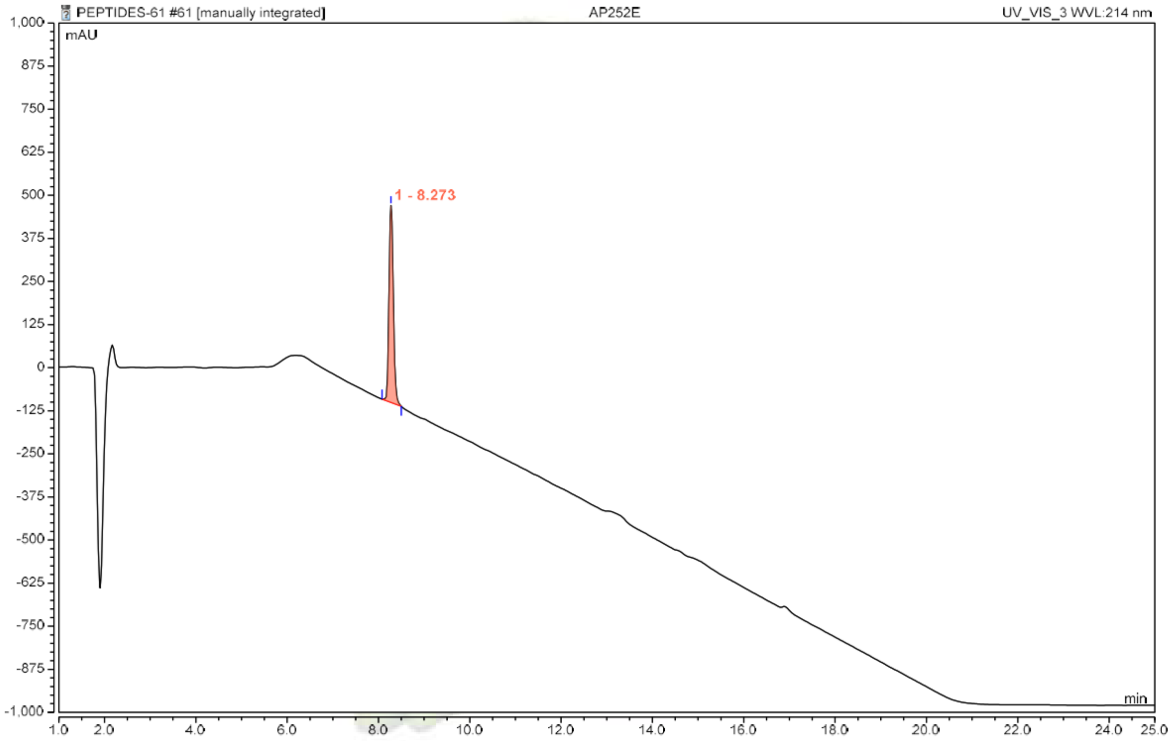

Fig. S15: HPLC trace of HPLC purified Novltex analogue **7** (gradient: 5–95% ACN in 25 min using A: 0.1% HCOOH in water, B: ACN).

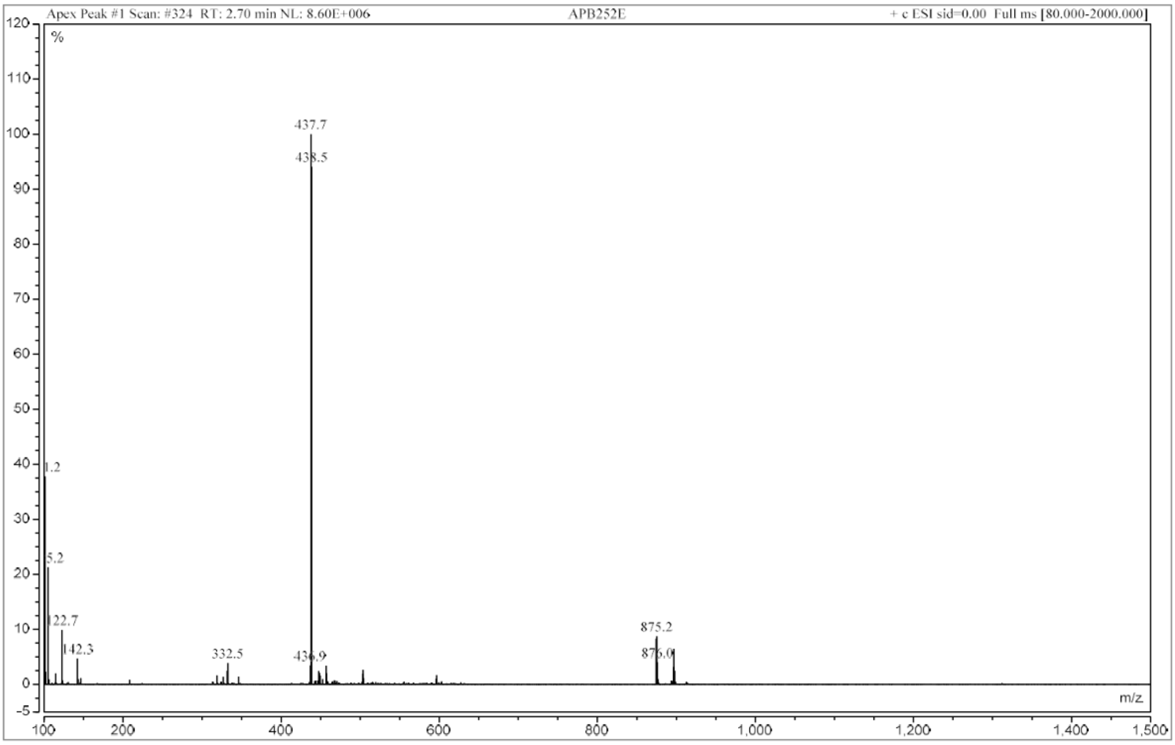

Fig. S16: MS spectra of HPLC purified Novltex analogue **7**. Exact Mass calcd. for $C_{43}H_{71}N_9O_{10}$ = 873.53, found $M+H^+$ = 875.2 and $M/2 + H^+$ = 437.7

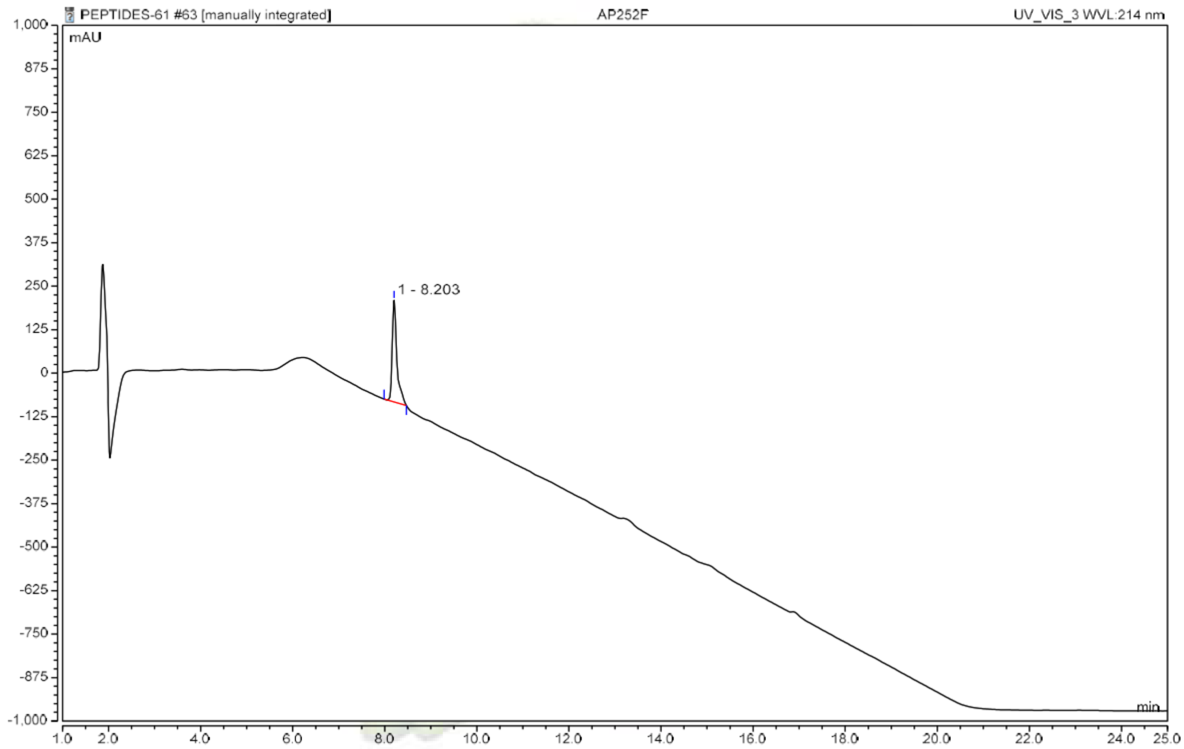

Fig. S17: HPLC trace of HPLC purified Novltex analogue **8** (gradient: 5–95% ACN in 25 min using A: 0.1% HCOOH in water, B: ACN).

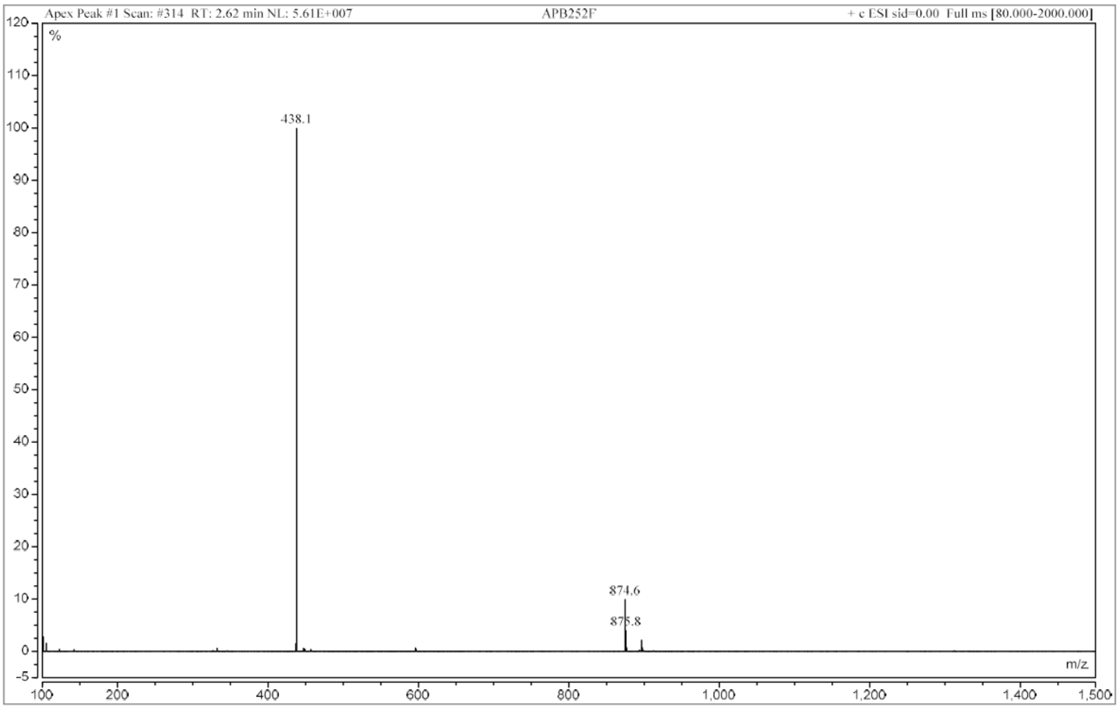

Fig. S18: MS spectra of HPLC purified Novltex analogue **8**. Exact Mass calcd. for $C_{43}H_{71}N_9O_{10}$ = 873.53, found $M+H^+$ = 874.6 and $M/2 + H^+$ = 438.1

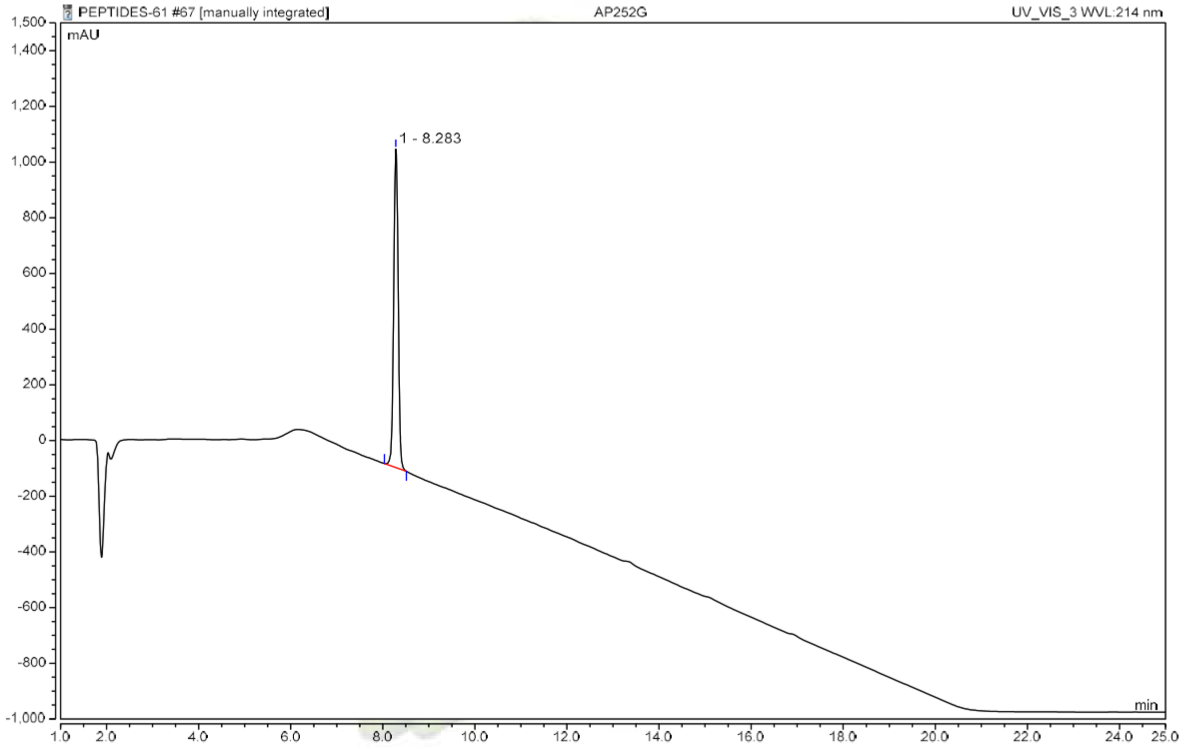

Fig. S19: HPLC trace of HPLC purified Novltex analogue **9** (gradient: 5–95% ACN in 25 min using A: 0.1% HCOOH in water, B: ACN).

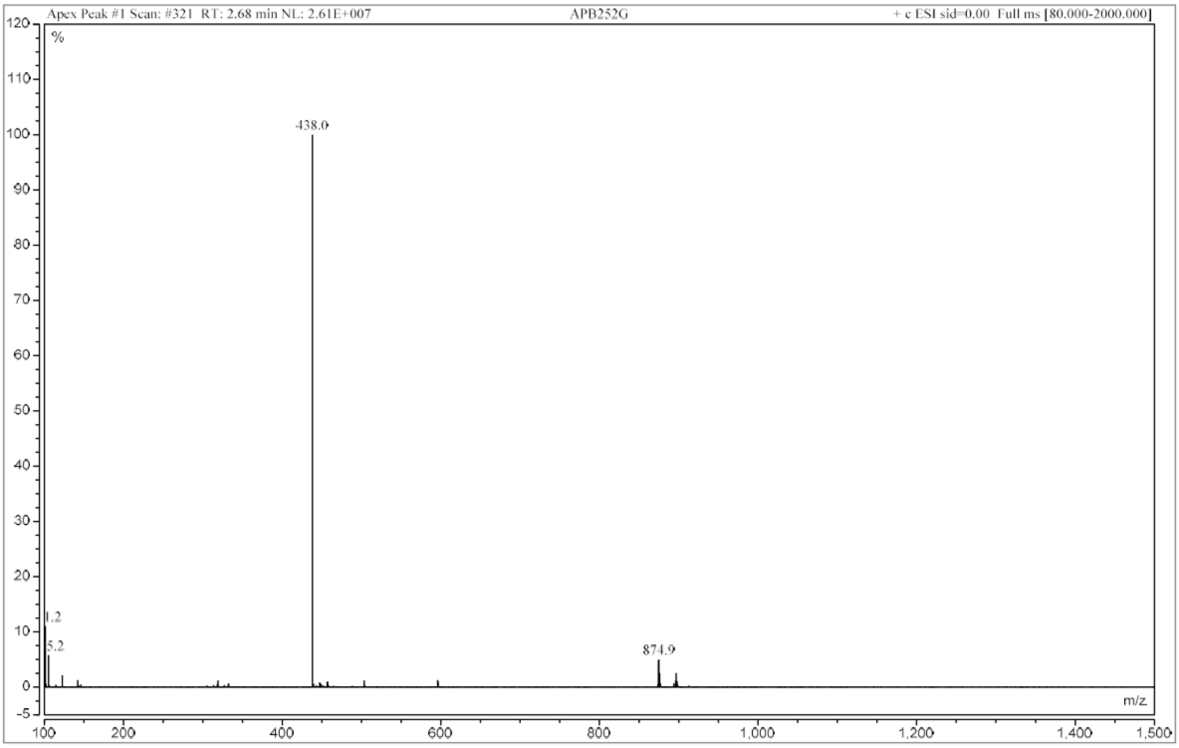

Fig. S20: MS spectra of HPLC purified Novltex analogue **9**. Exact Mass calcd. for $C_{43}H_{71}N_9O_{10}$ = 873.53, found $M+H^+$ = 874.9 and $M/2 + H^+$ = 438.0

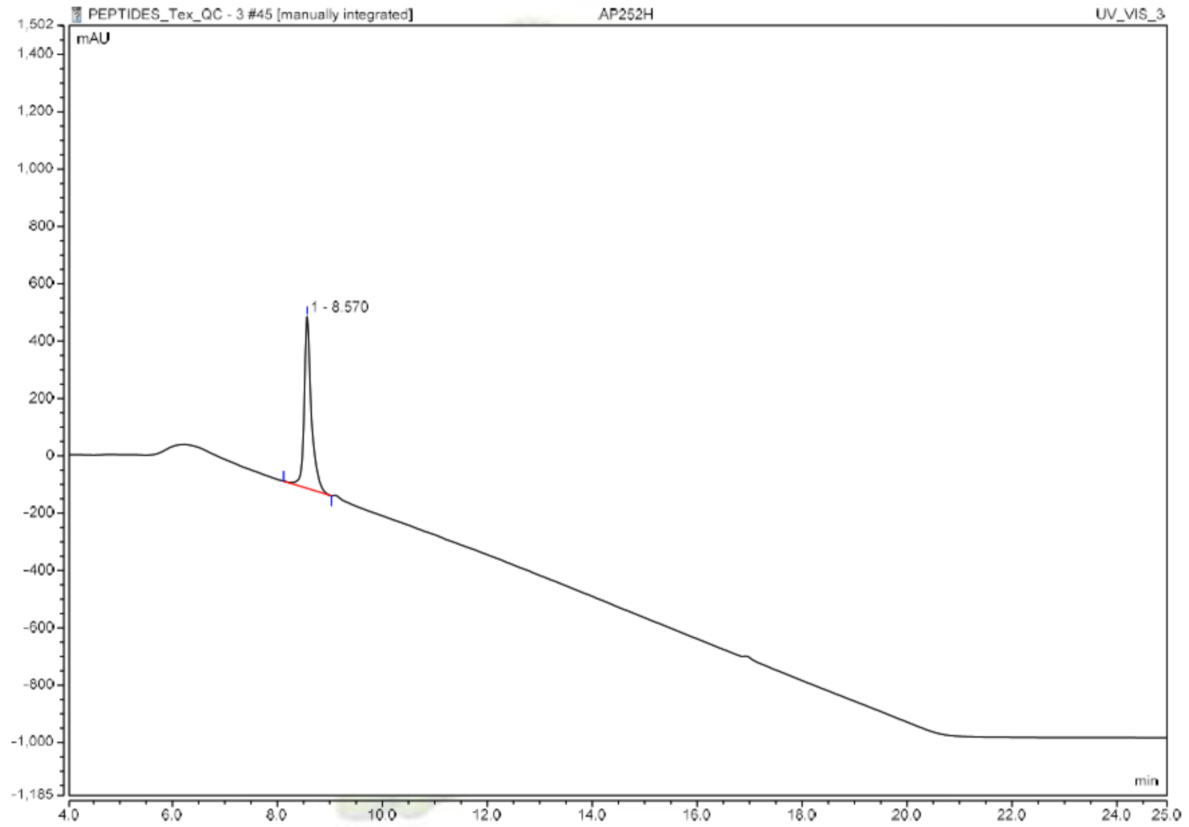

Fig. S21: HPLC trace of HPLC purified Novltex analogue **10** (gradient: 5–95% ACN in 25 min using A: 0.1% HCOOH in water, B: ACN).

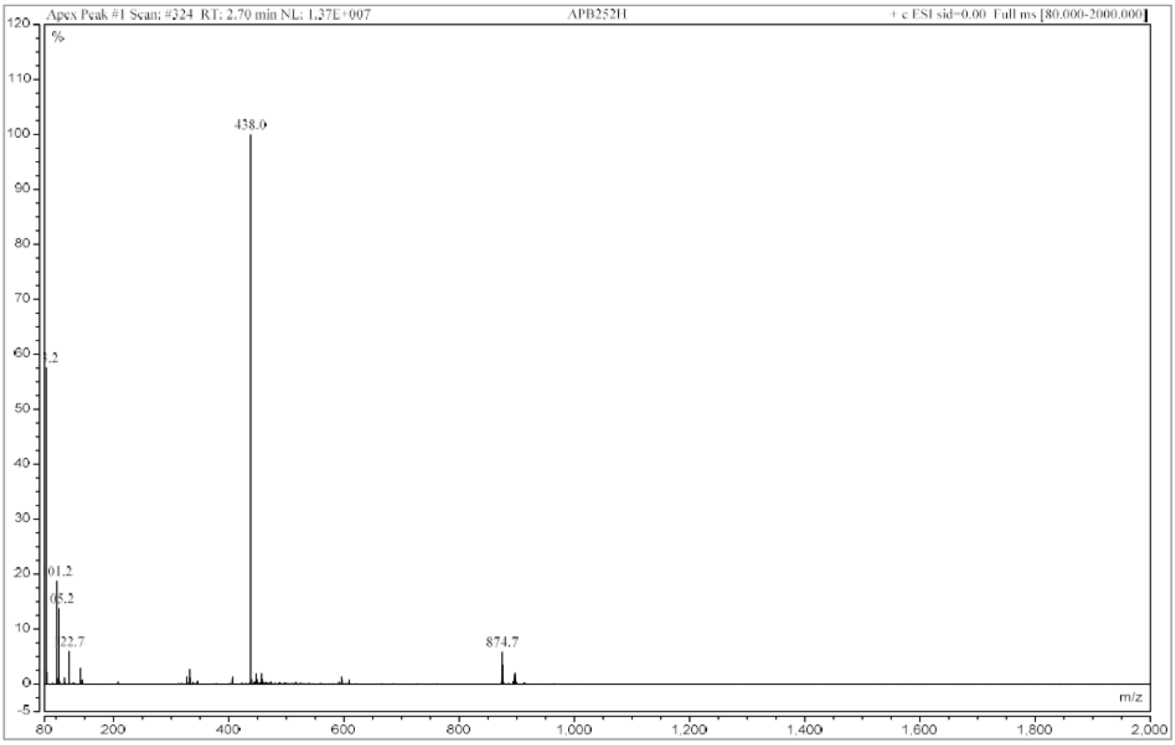

Fig. S22: MS spectra of HPLC purified Novltex analogue **10**. Exact Mass calcd. for $C_{43}H_{71}N_9O_{10}$ = 873.53, found $M+H^+$ = 874.7 and $M/2 + H^+$ = 438.0

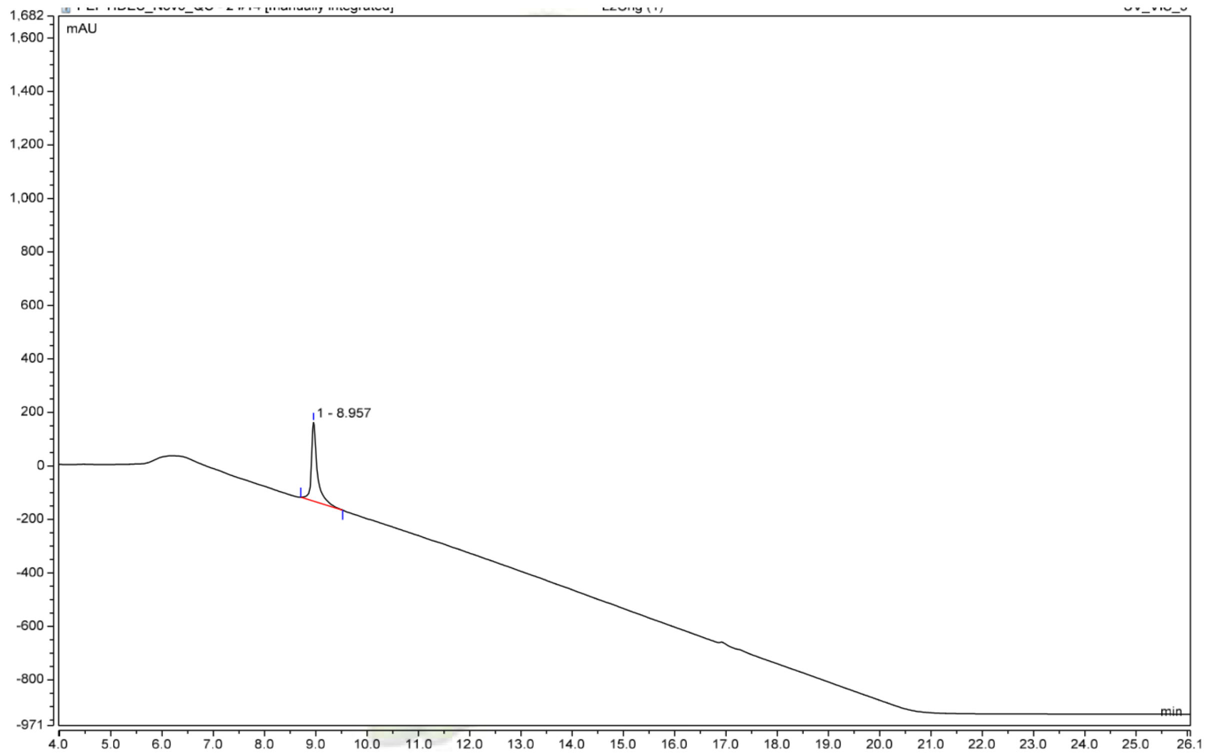

Fig. S23: HPLC trace of HPLC purified Novltex analogue **11** (gradient: 5–95% ACN in 25 min using A: 0.1% HCOOH in water, B: ACN)

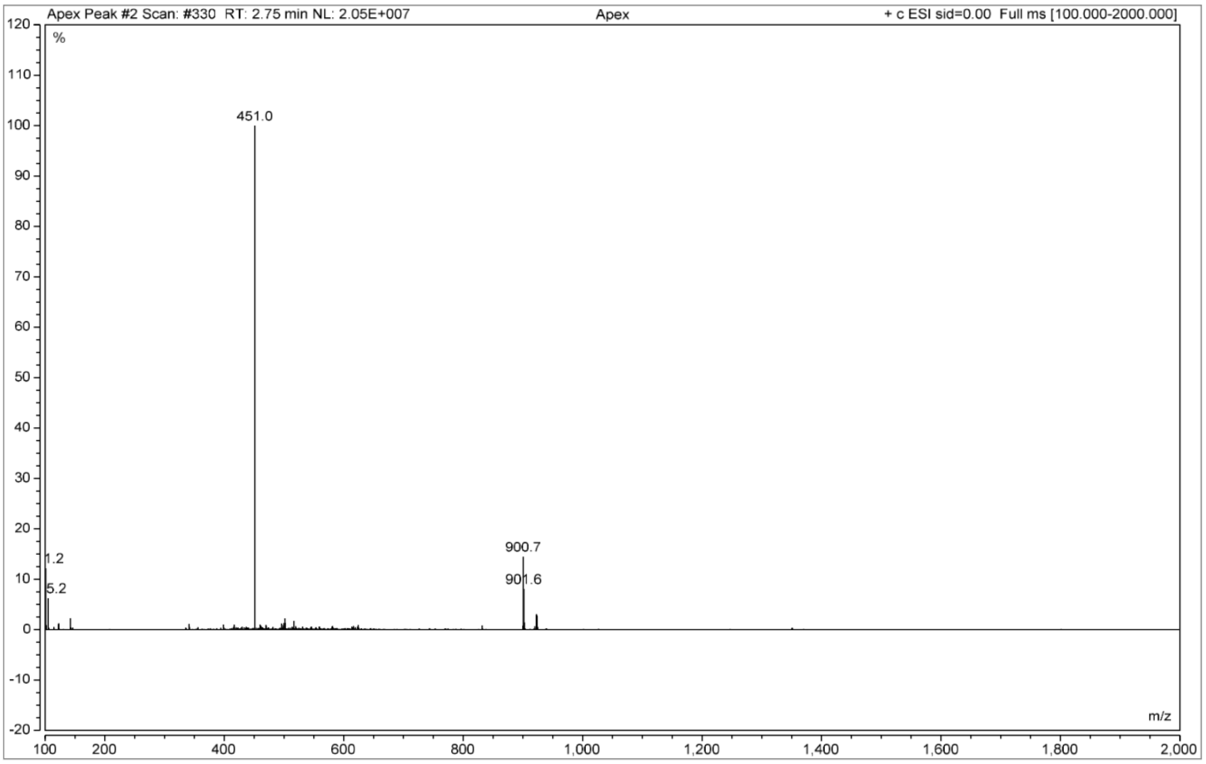

Fig. S24: MS spectra of HPLC purified Novltex analogue **11**. Exact mass calcd. for $C_{45}H_{73}N_9O_{10}$= 899.54, found M + H^+ = 900.7, M/2 + H^+ = 451.0.

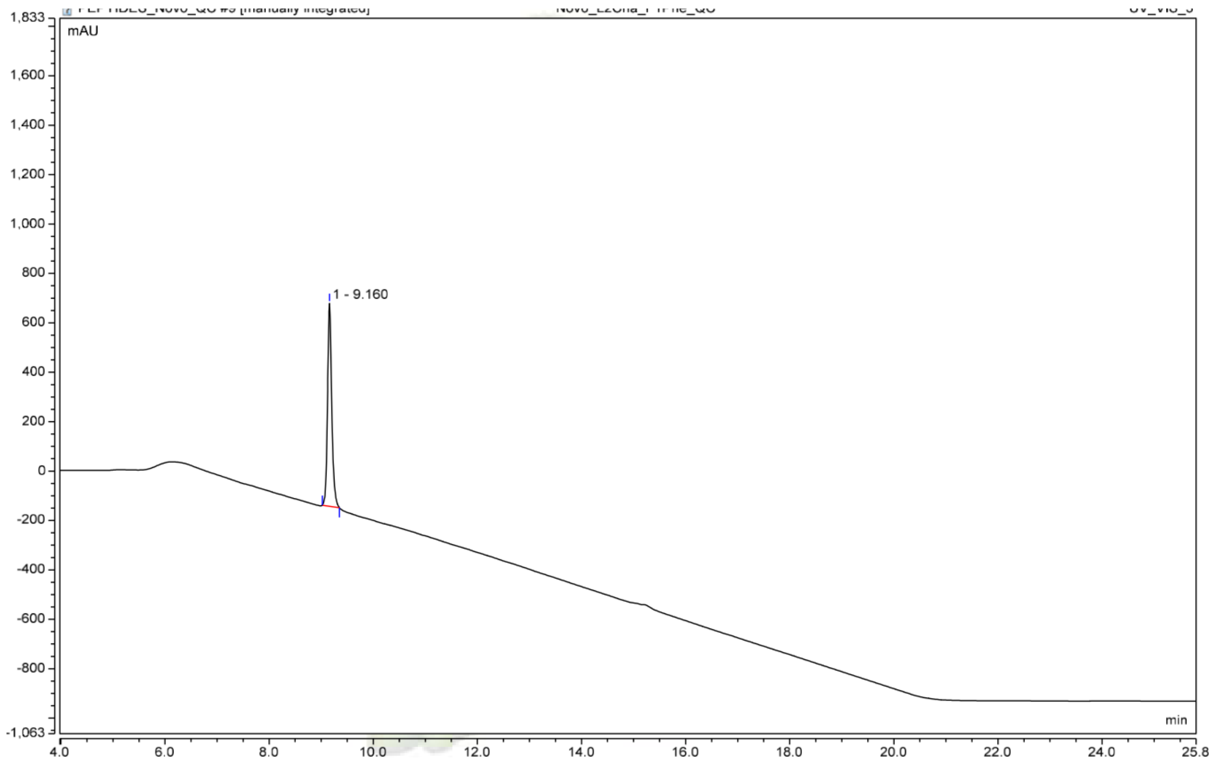

Fig. S25: HPLC trace of HPLC purified Novltex analogue **12** (gradient: 5–95% ACN in 25 min using A: 0.1% HCOOH in water, B: ACN)

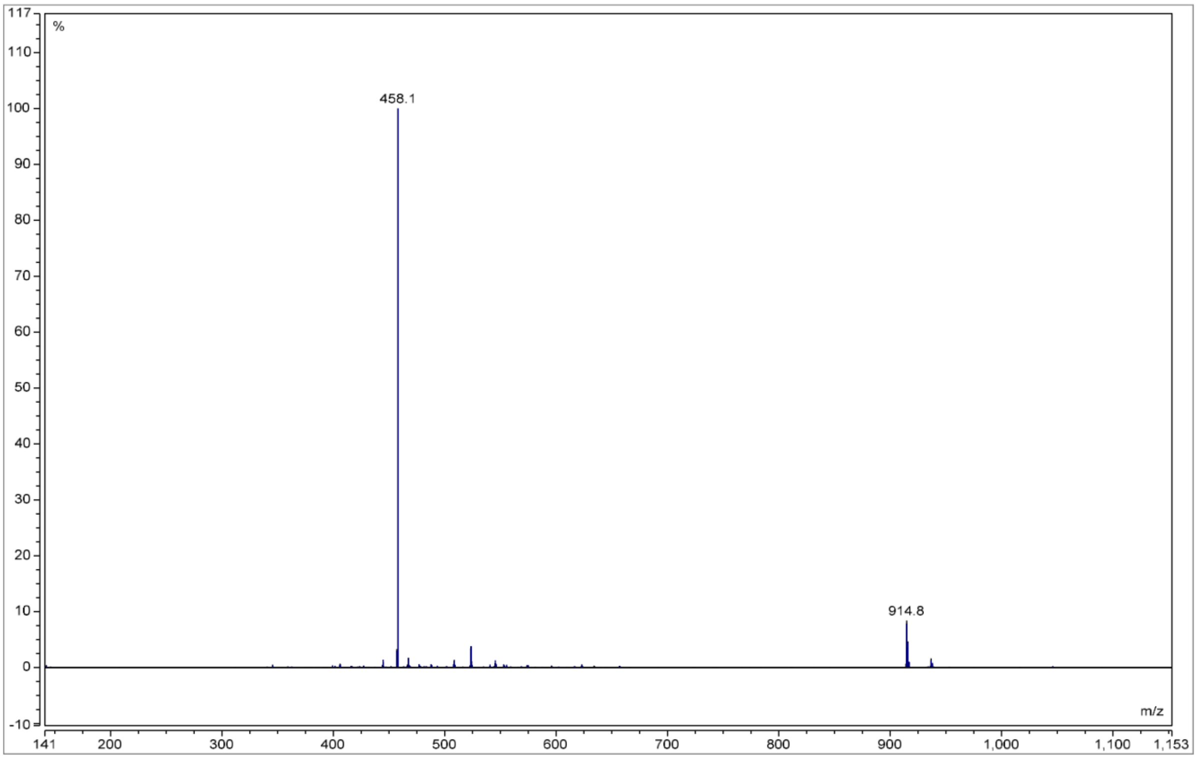

Fig. S26: MS spectra of HPLC purified Novltex analogue **12**. Exact mass calcd. for $C_{46}H_{75}N_{9}O_{10}$= 913.56, found M + H^{+} = 914.8, M/2 + H^{+} = 458.1.

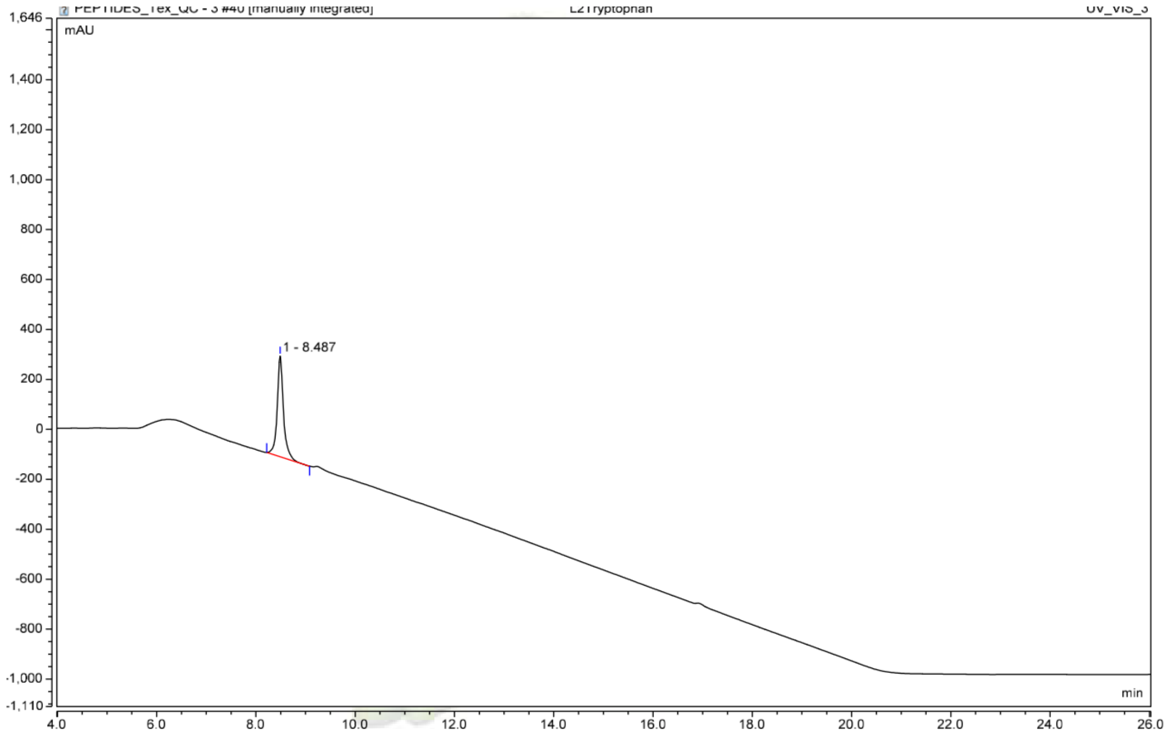

Fig. S27: HPLC trace of HPLC purified Novltex analogue **13** (gradient: 5–95% ACN in 25 min using A: 0.1% HCOOH in water, B: ACN)

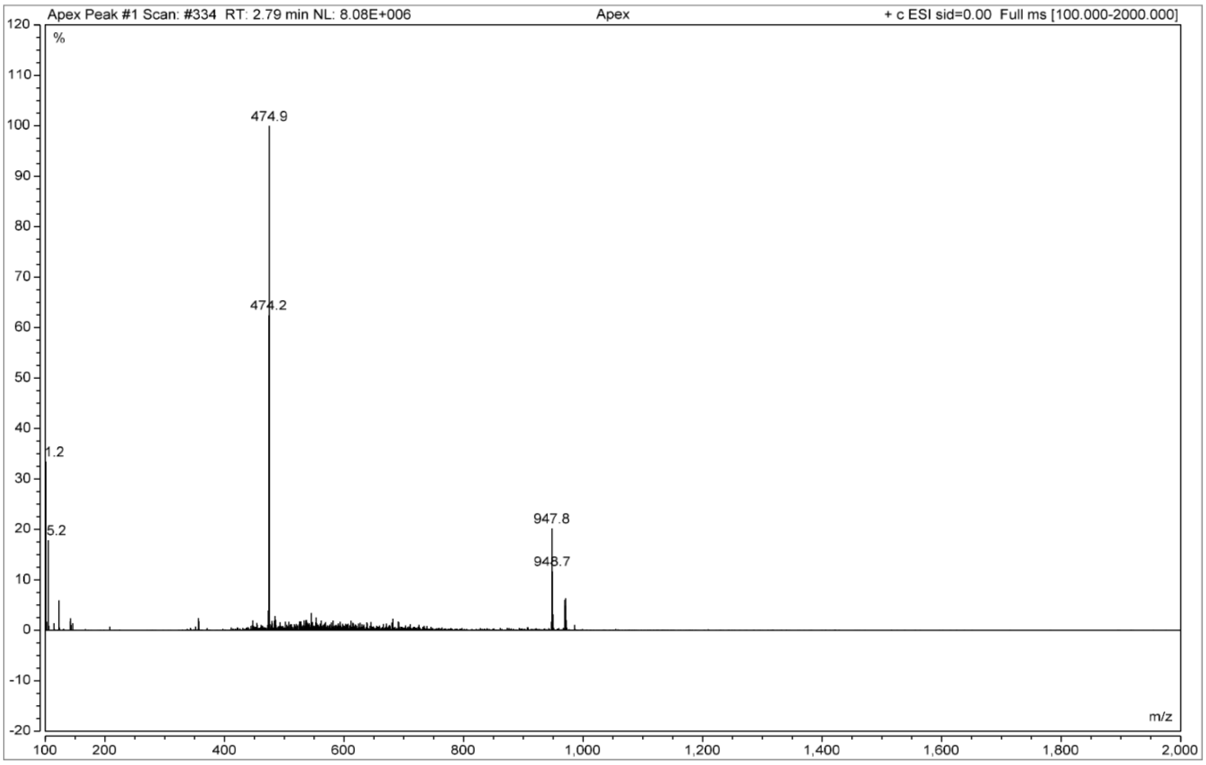

Fig. S28: MS spectra of HPLC purified Novltex analogue **13**. Exact mass calcd. for $C_{49}H_{71}N_9O_{10}$= 946.52, found M + H$^+$ = 947.8, M/2 + H$^+$ = 974.2.

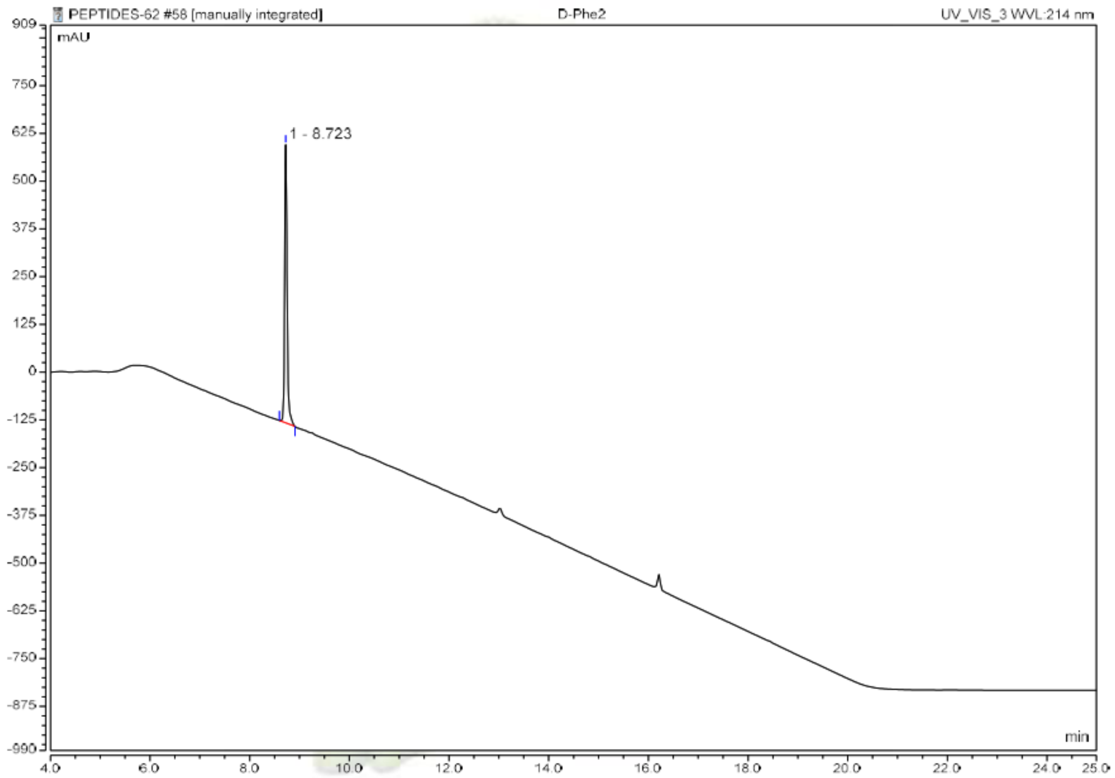

Fig. S29: HPLC trace of HPLC purified Novltex analogue **14** (gradient: 5–95% ACN in 25 min using A: 0.1% HCOOH in water, B: ACN)

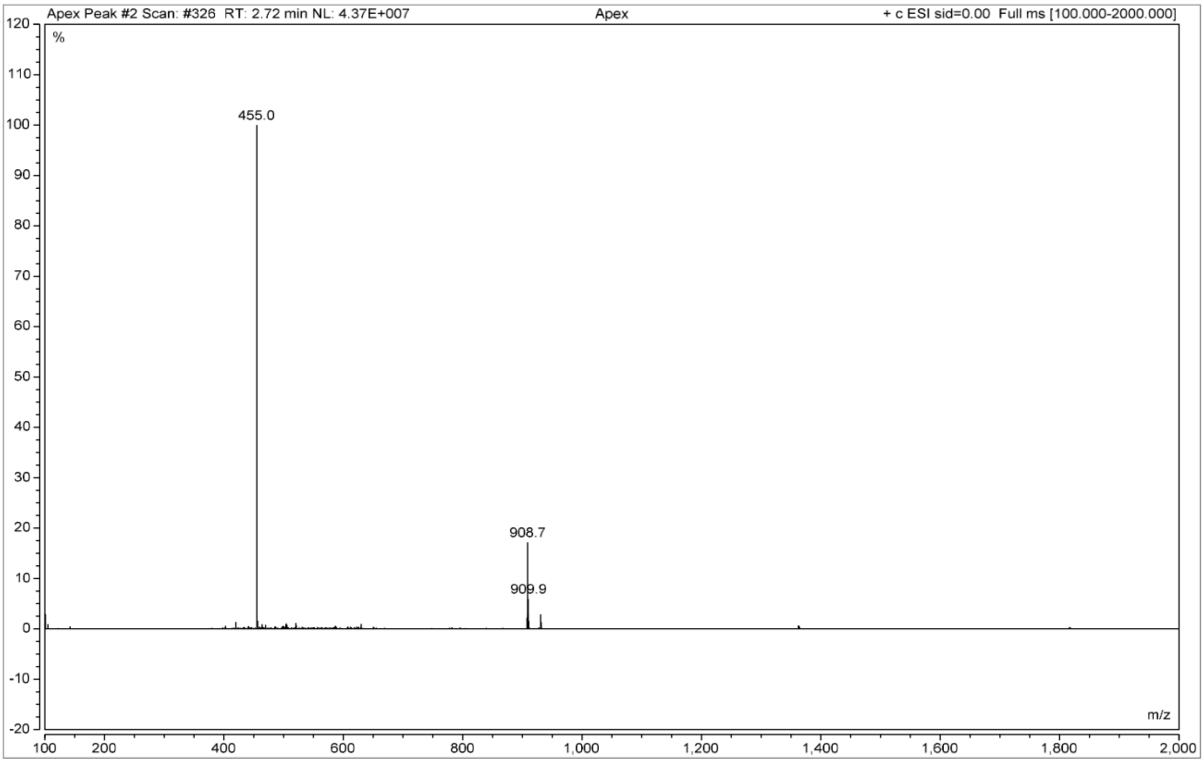

Fig. S30: MS spectra of HPLC purified Novltex analogue **14**. Exact mass calcd. for $C_{46}H_{69}N_9O_{10}$= 907.51, found M + H^+ = 908.7, M/2 + H^+ = 455.0.

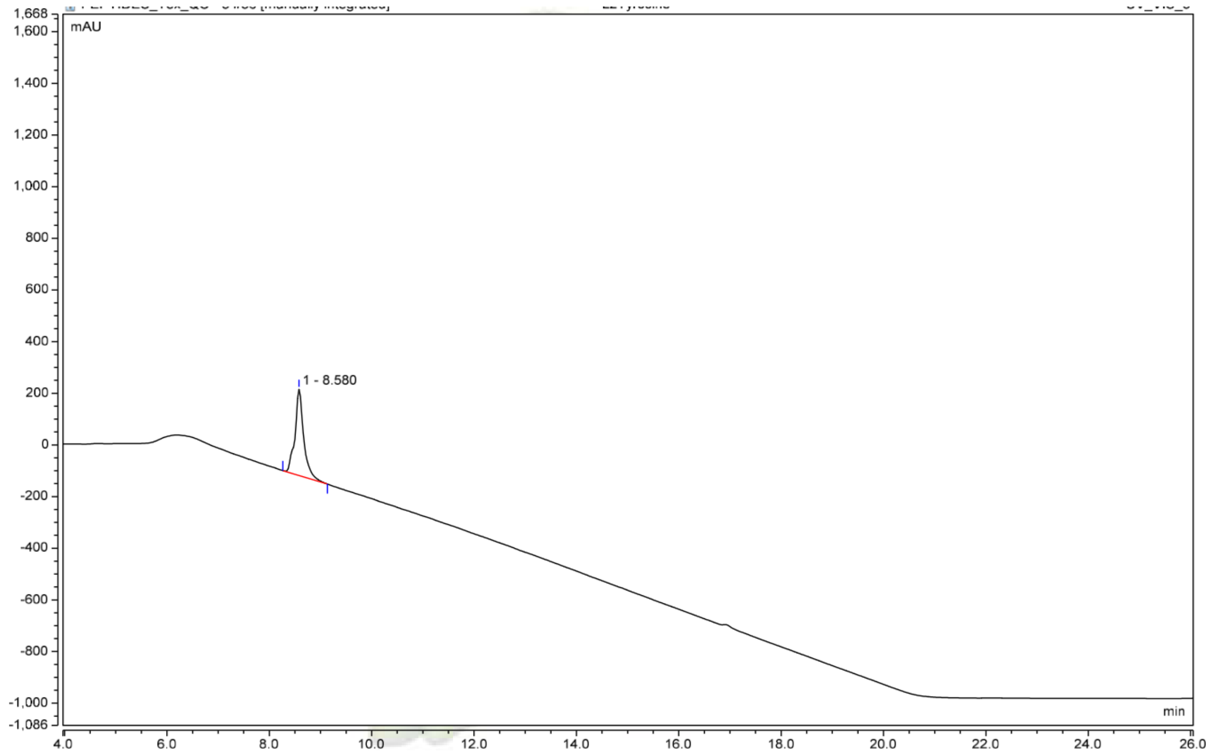

Fig. S31: HPLC trace of HPLC purified Novltex analogue **15** (gradient: 5–95% ACN in 25 min using A: 0.1% HCOOH in water, B: ACN)

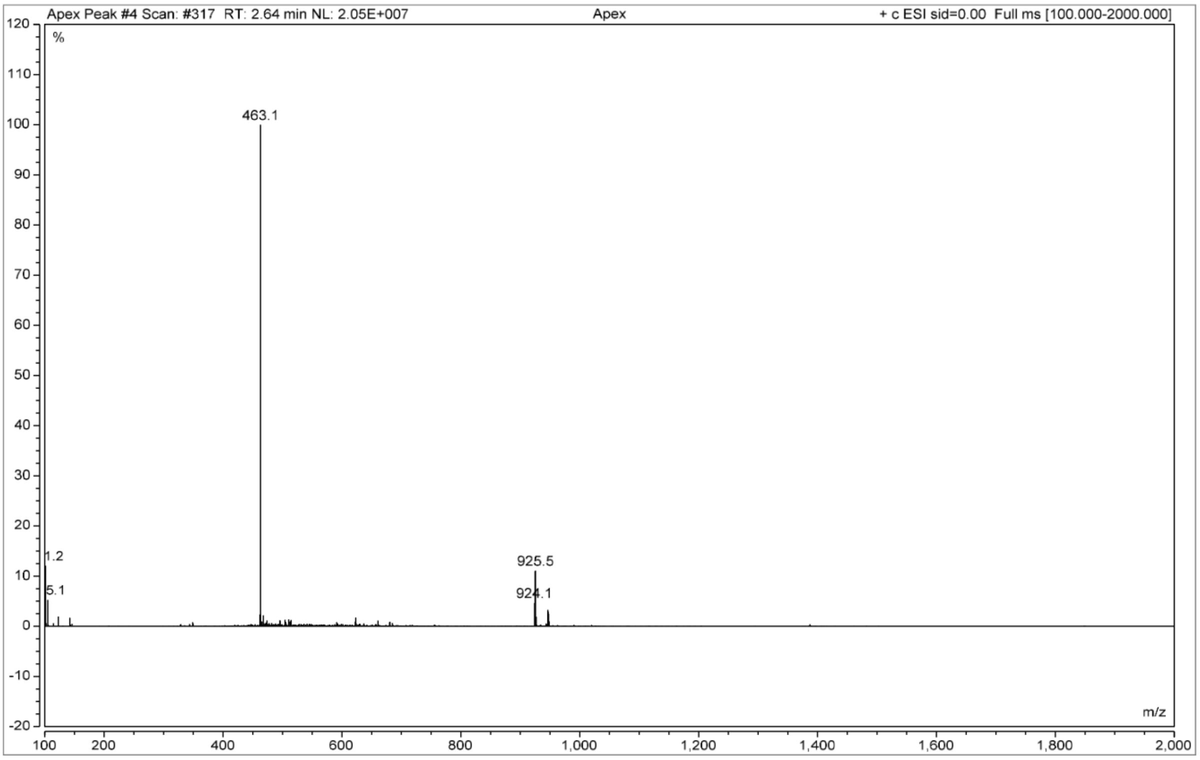

Fig. S32: MS spectra of HPLC purified Novltex analogue **15**. Exact mass calcd. for $C_{46}H_{69}N_9O_{11}$= 923.51, found M + H$^+$ = 925.5, M/2 + H$^+$ = 463.1.

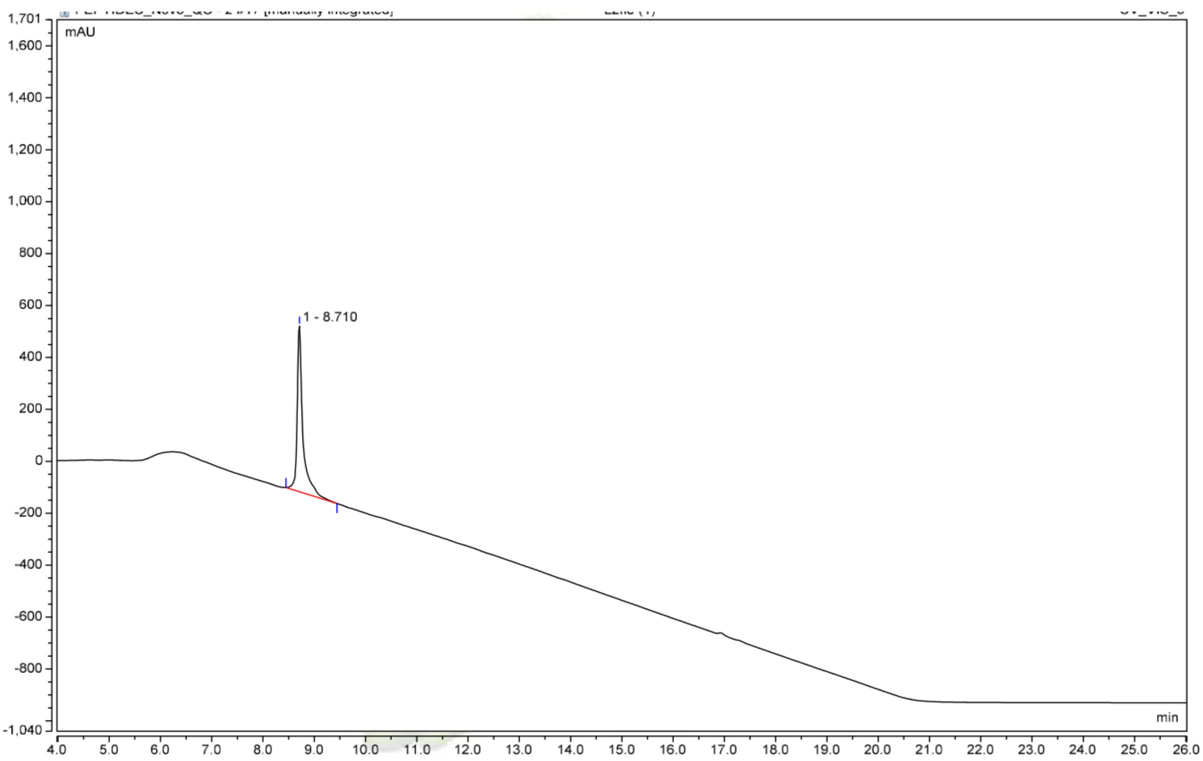

Fig. S33: HPLC trace of HPLC purified Novltex analogue **16** (gradient: 5–95% ACN in 25 min using A: 0.1% HCOOH in water, B: ACN)

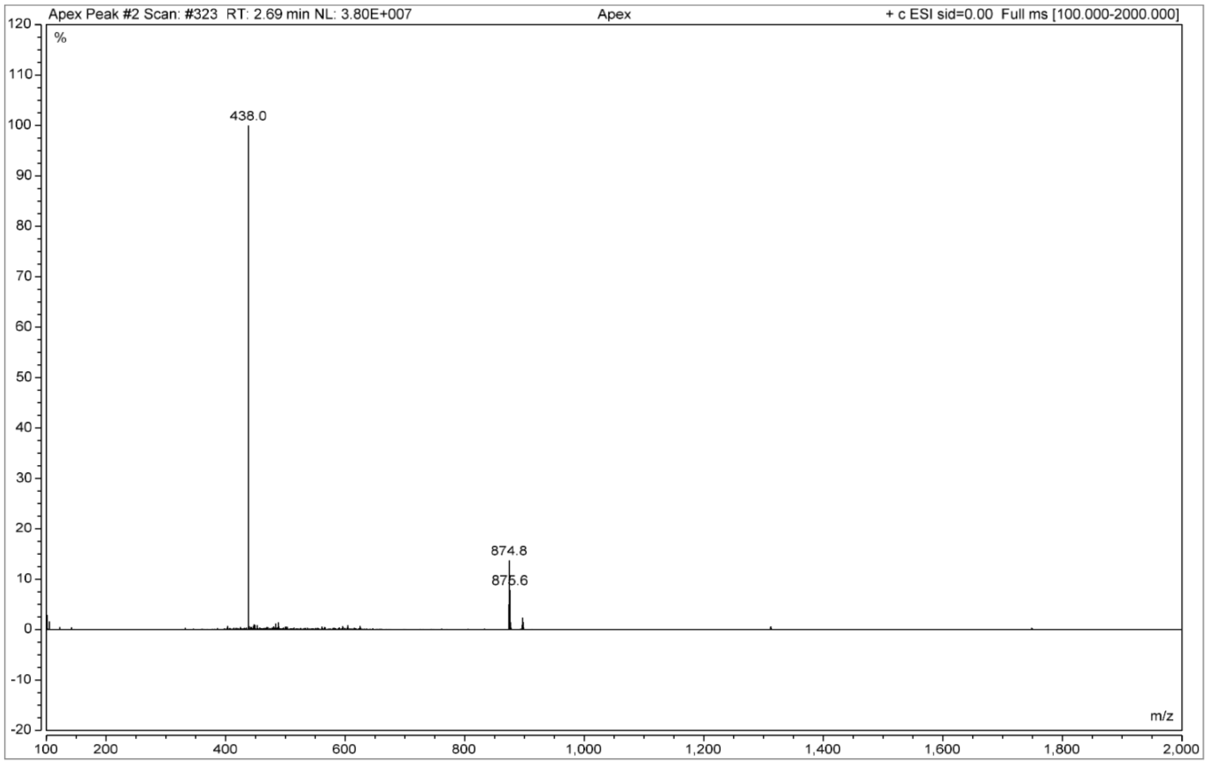

Fig. S34: MS spectra of HPLC purified Novltex analogue **16**. Exact mass calcd. for $C_{43}H_{71}N_9O_{10}$= 873.53, found M + H^+ = 874.8, M/2 + H^+ = 438.0.

VI. NMR data

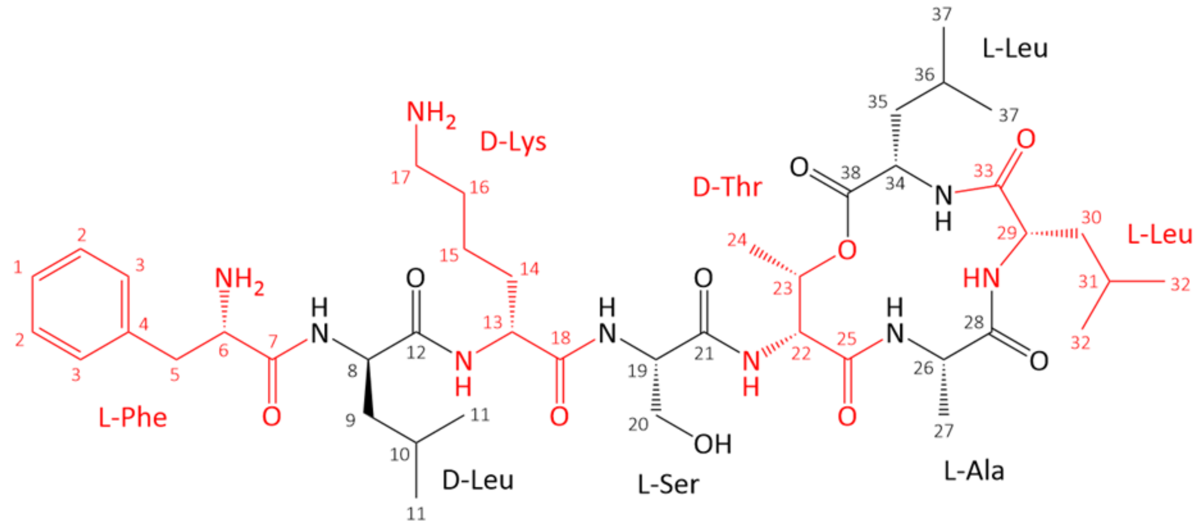

Fig. S35: Chemical structure of Novltex analogue **4** with the labels for the spectral assignments based on the spectra recorded and the data tabulated in **Table 3**.

	Atoms number	δ_H (*J* in Hz)	δ_C
Phe	1	7.23, m	126.78
	2	7.29, m	129.32
	3	7.23, m	128.35
	4	-	129.37
	5	2.93, m	40.33, 40.51*
	6	3.98, m	54.47
	7	-	163.27, 170.53, 171.28, 171.57, 171.84*
	NH_2-6	8.16, s	-
Leu	8	4.17, m	52.03
	9	1.24, m	29.02
	10	1.33, m	23.14
	11	0.78, d (6.4)	21.19
	12	-	163.27, 170.53, 171.28, 171.57, 171.84*
	NH-8	8.51, d (7.5)	-
Lys	13	4.34, m	50.83
	14	1.80, m	30.85
	15	1.33, m	22.44
	16	1.50, m	26.32
	17	2.72, t (7.4)	38.65
	18	-	163.27, 170.53, 171.28, 171.57, 171.84*
	NH-13	8.26, d (8.5)	-
Ser	19	4.35, m	56.11
	20	3.70, 3.84, dd (3.4)	62.05
	21	-	171.54
	NH-19	8.00, d (6.5)	-
Thr	22	4.65, dd (10.0, 2.1)	55.24
	23	5.26, qd (6.4, 2.1)	70.67
	24	1.04, d (6.4)	15.32
	25	-	168.11
	NH-22	8.80, d (10.0)	-
Ala	26	3.87, m	51.72
	27	1.25, d (7.5)	16.90
	28	-	171.95
	NH-26	7.97, d (5.0)	-
Leu	29	4.34, m	52.24
	30	1.50, m	23.96
	31	1.50, m	21.69
	32	0.88, (6.0)	22.65
	33	-	163.27, 170.53, 171.28, 171.57, 171.84*
	NH-29	8.20, d (10.5)	-
Leu	34	4.34, m	52.58
	35	1.50, m	24.41, 24.69*
	36	1.50, m	24.41, 24.69*
	37	0.83#	21.50
	38	-	163.27, 170.53, 171.28, 171.57, 171.84*
	NH-34	8.54, d (10.2)	-

Table. S3: NMR spectral assignments for Novltex **4**. Chemical shifts and *J* couplings are given with ± 0.01 ppm and ± 0.2 Hz accuracies, respectively. -, * and # denote not applicable, ambiguous assignment, and *J* coupling multiplicity not determined, respectively. s, d, t, q and m refer to the multiplicity of the corresponding resonances and stand for singlet, doublet, triple, quadruplet and multiplet, respectively.

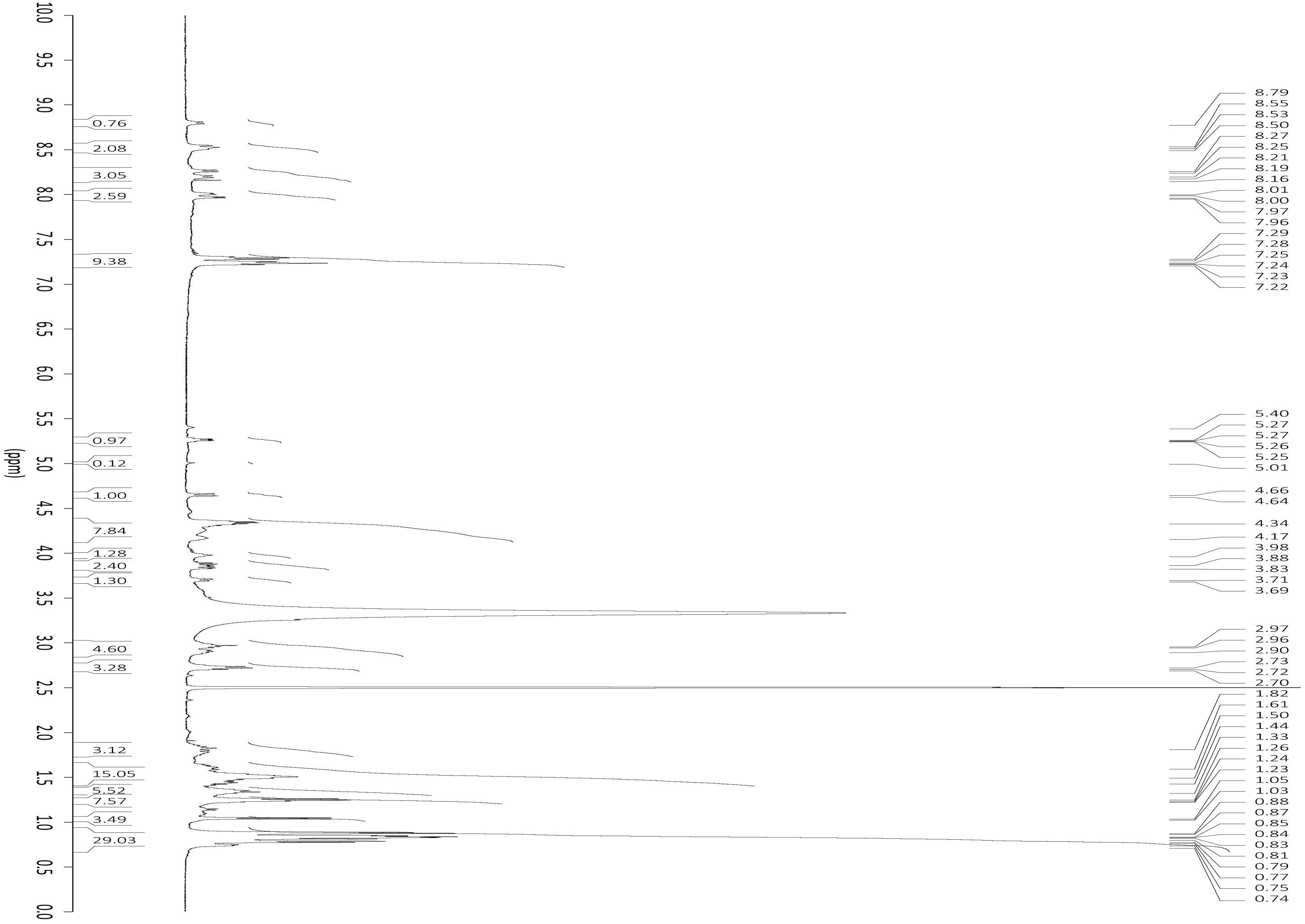

Fig. S36: ^{1}H spectrum of Novltex **4** (3 mM) in DMSO-d_6. The large broad signal at 3.3 ppm is water.

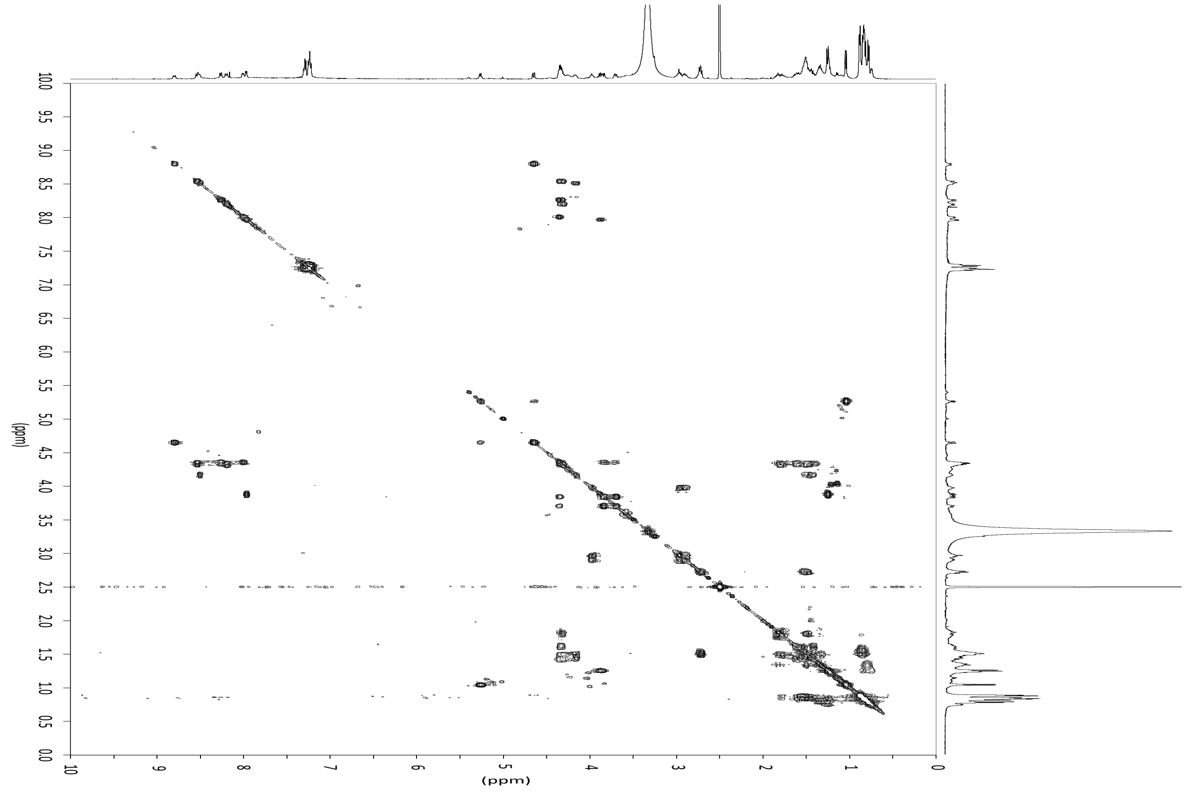

Fig. S37: ^{1}H ^{1}H COSY spectrum of Novltex **4** (3 mM) in DMSO-d_6.

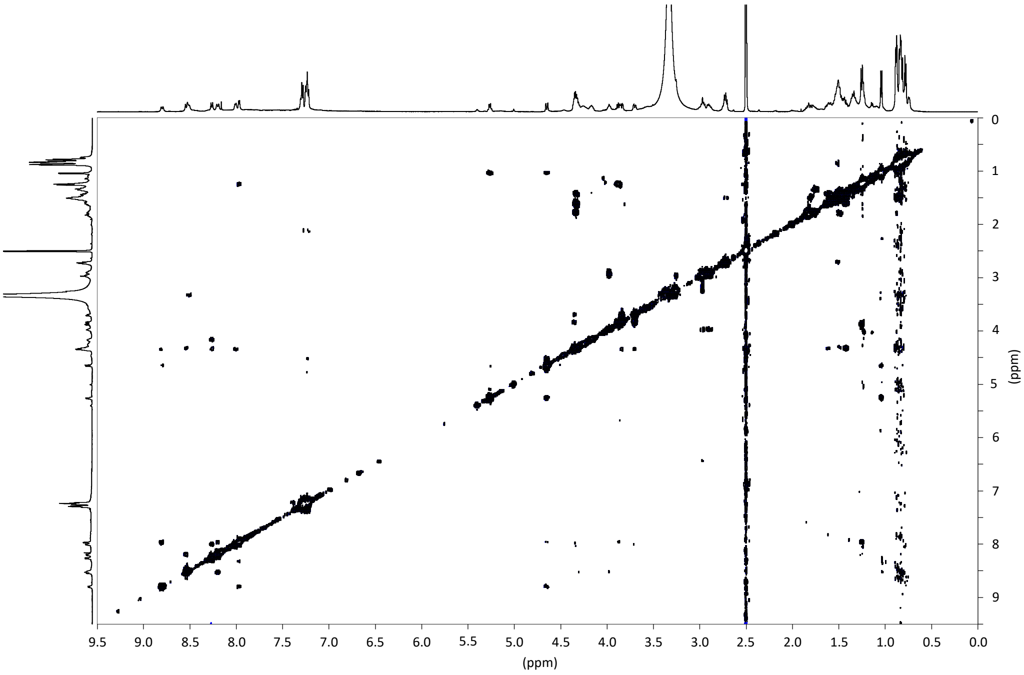

Fig. S38: ^{1}H ^{1}H NOESY spectrum of Novltex **4** (3 mM) in DMSO-d_6.

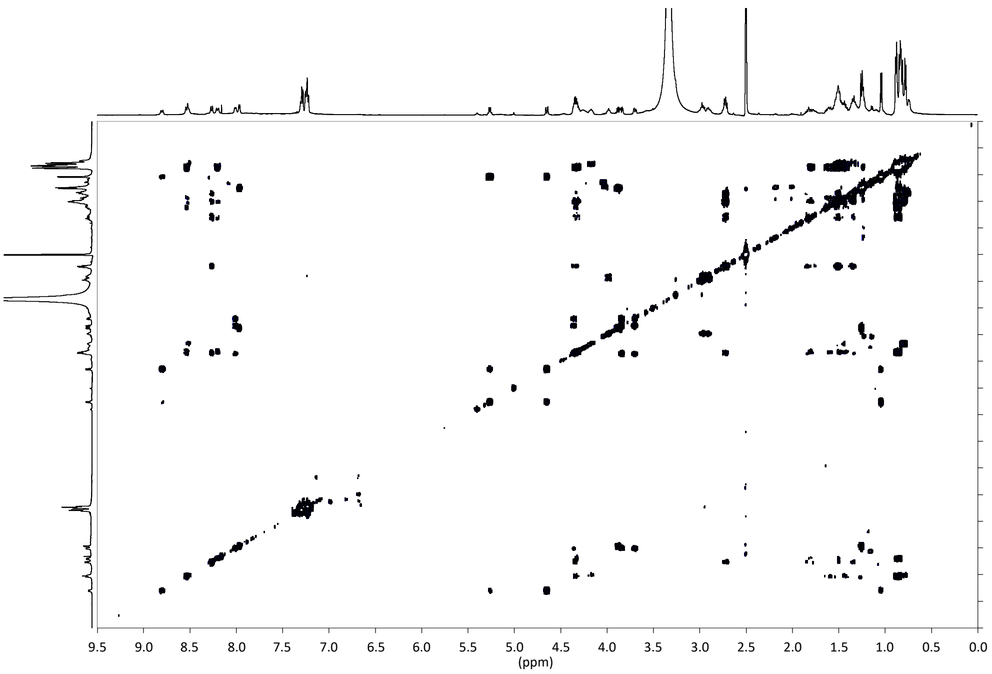

Fig. S39: ^{1}H ^{1}H TOCSY spectrum of Novltex **4** (3 mM) in DMSO-d$_6$.

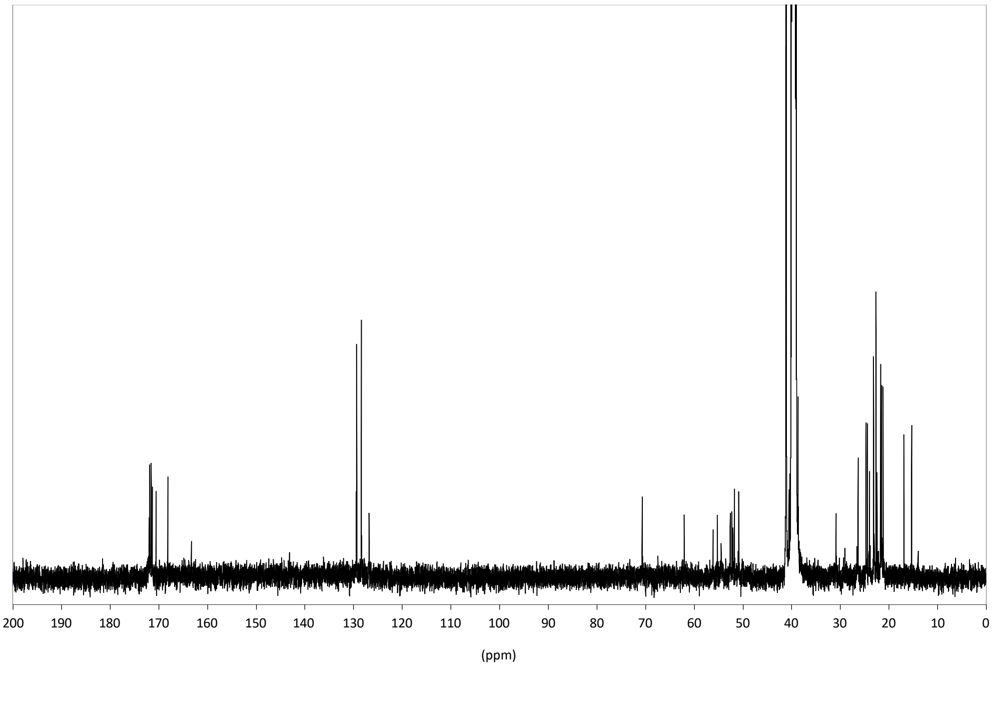

Fig. S40: ^{13}C{^{1}H} spectrum of Novltex **4** (3 mM) in DMSO-d_6.

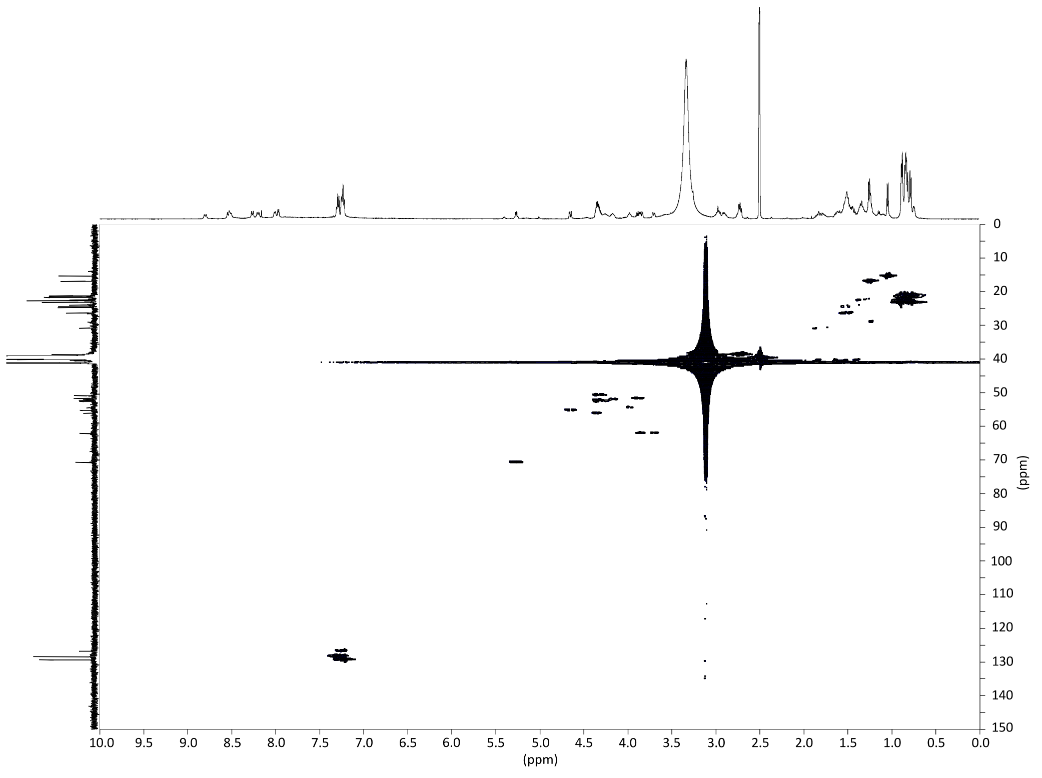

Fig. S41: ^{1}H ^{13}C HSQC spectrum of Novltex **4** (3 mM) in DMSO-d_6.

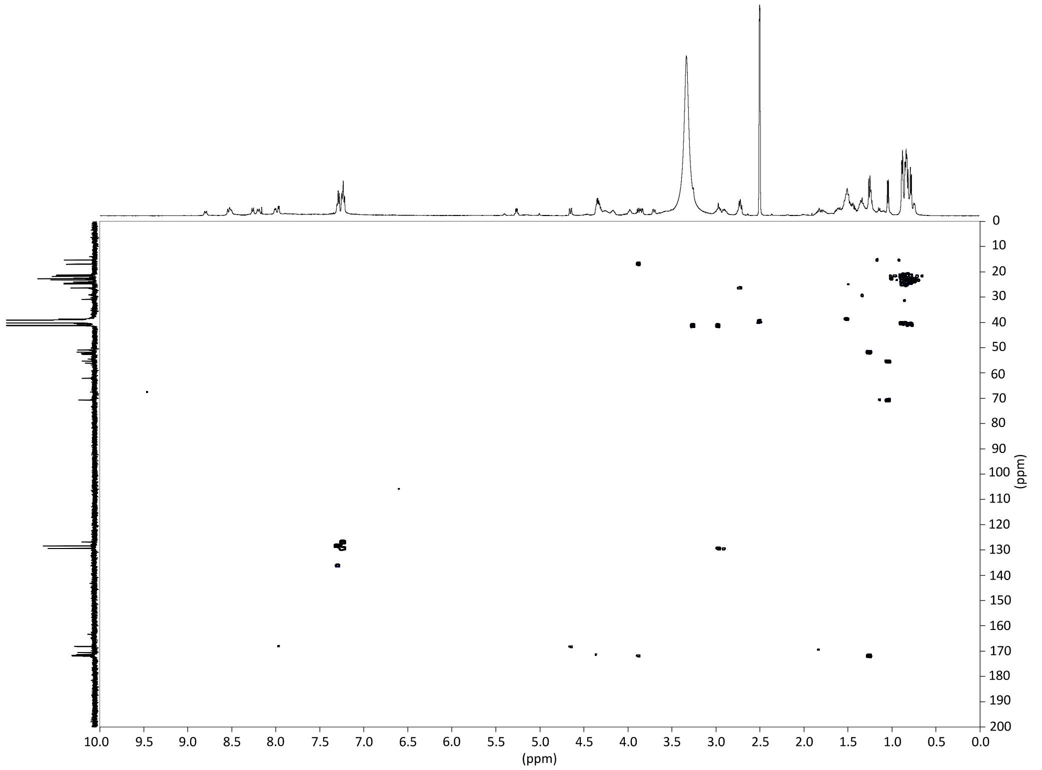

Fig. S42: ^{1}H ^{13}C HMBC spectrum of Novltex **4** (3 mM) in DMSO-d_6.

VII. MIC testing

MICs were performed as previously reported [4]. For MIC testing all peptides were dissolved in DMSO. Bacterial cultures were grown overnight on Mueller-Hinton Agar (MHA) plates and adjusted to a final inoculum of 10^5 – 10^6 CFU/mL. 100 µl of inoculum in Meuller-Hinton broth (MHB) was mixed with equal volume of peptides (dissolved in MHB) at 2x their concentration in a 96 well plate. For daptomycin MIC determination, the same protocol was followed with the addition of 50 mg/L calcium chloride to the medium. MIC values were determined in the media containing polysorbate 80 (0.002%, v/v) to prevent non-specific adsorption of the peptides to plastic surfaces. The final peptides concentrations ranged from 0.0625 – 32 µg/mL. The 96 well plates were then incubated at 37 °C for 24 h. All the experiments were performed in two independent duplicates and the MIC was determined as the lowest concentration in which no visible growth was observed.

**Antibiotics**	**MIC break points (µg/mL)**
Vancomycin	≤2
Linezolid	4
Cefotaxime	≤8
Levofloxacin	≤1
Ampicillin	0.6 -1
Daptomycin	1

Table. S4: Susceptibility cut-off values (MIC breakpoints) for various clinical antibiotics.

VIII. Time dependent killing of bacteria and resistance studies by analogue 12

Time-kill kinetics against MRSA ATCC 33591 was carried out on MHB. Cultures were grown overnight on MHA plates and adjusted to a final inoculum of 10^5 – 10^6 CFU/mL in MHB (containing 0.002% v/v, polysorbate 80) with Novltex analogue 12 and vancomycin at a final concentration of 2.5 µg/mL and 5 µg/mL respectively. The tubes were then incubated at 37 °C. 100 µl of cell suspension was withdrawn at various time points (0, 1, 2, 4, 6, 24 h), serially diluted and plated onto MHA plates and incubated for 24 h at 37 °C. Colony counting too numerous to count (>300 colonies) was taken as 10^{10} CFU. Average values from two independent duplicates experiments are reported.

Resistance evaluations were conducted following previously described protocols[5].

IX. Cytotoxicity Assay

HepG2 (ATCC #HB-8065), HEK293T (ATCC #CRL-3216), A549 () ATCC # CCL-185), and primary dermal fibroblasts (PromoCell #C-12302), were cultured in DMEM supplemented with 10% heat-inactivated fetal bovine serum. All cell types were cultured according to ATCC recommended guidelines and maintained at 37 °C in 5% CO_2 humidified incubator. All the cell lines were verified as mycoplasma free using MycoGuard™ Mycoplasma PCR Detection Kit (GeneCopoeia #MP004). Cells (5×10^3/well) were seeded in 96-well plates and allowed them to adhere on culture plates. The following

day, cells were treated in triplicates with increasing concentrations of Novltex analogue **12,** as indicated in the corresponding figure and the figure legend (Figure 4A-C). Cells were treated with 1 µM Staurosporine (MedChemExpress #HY-15141) as a positive kill control. Cell viability assessed after 24 h or 48 h incubation using an MTS-based CellTiter 96® AQueous One Solution Cell Proliferation Assay (Promega #G3582, Southampton UK) as per manufacturer's instructions. Absorbance was recorded at 490 nm. Relative cell viability was determined by subtracting the blank (cell culture medium and MTS only) from individual values before normalizing against the untreated control.

X. Hemolysis Assay

Anonymized human blood samples were received from the Health Sciences Authority, Singapore and used as per the institutional guidelines and approved by the Institutional Review Board (IRB-2023-1019) of Nanyang Technological University Singapore. Freshly received blood was centrifuged at 3000 rpm for 10 mins at 4 °C and washed two times with 20 mM PBS (phosphate buffered saline, pH 7.0) to prepare erythrocytes or RBCs. Novltex analogue 12 was prepared at 2× in the same buffer, diluted 1:1 with 8% v/v blood preparation so that the final concentration of the peptide remained 1.95, 3.9, 7.81, 15.62, 31.25, or 62.5 µg/mL. The blood preparation was treated with equivalent amount of PBS alone or 0.1% (v/v) Triton X-100 as negative and positive controls. The mixture was transferred to a 96-well microplate and incubated at 37 °C for 1 hour. After incubation, the mixture was transferred to microtubes and centrifuged at 3000 rpm for 3 min to collect the supernatant. The supernatant was transferred to a new 96-well microplate and the absorbance was measured at 576 nm in the Infinite M200 monochromator microplate reader (Tecan Group Ltd., Switzerland). The percentage hemolysis was calculated using the equation:

$$\% \text{ Hemolysis} = (A0 - Amin) / (Amax - Amin) \times 100$$

where A0 is the observed absorbance, Amin is the average absorbance of 4% v/v blood without treatment, Amax is the average absorbance of 8% v/v blood diluted 1:1 with 0.1% Triton X-100.

XI. Cytoplasmic membrane potential $DiSC_3(5)$ assay

MRSA DM21455 bacterial cells were grown overnight in MHB and washed with 1× HEPES buffer containing 5 mM glucose. The cell concentration was adjusted to OD 0.4. After addition of 10 µM $DiSC_3(5)$ dye, the mixture was incubated for 1 h in dark at room temperature. Next, 600 µL of the dye-loaded cell suspension was transferred to a 1.5 mL tube and Novltex analogue **12** at different final concentrations (1×, 2×, 4×, 8×, and 16× MIC) were added. The mixture was transferred to a quartz cuvette after incubating for further 1 h in the dark at room temperature. The fluorescence intensity was monitored using a Quanta Master spectrofluorimeter (Photon Technology International, NJ, USA) at excitation 622 nm and emission 670 nm. Triton x-100 (1% v/v) was used as a positive control to obtain maximum change in fluorescence intensity.

XII. Lipid II binding determination of analogue 12

Previously reported methods were used to determine the lipid II binding of analogue **12**[6,7]. The lipid II used in this study was prepared by chemical synthesis and its preparation and purification has been previously reported[1].

A: An overnight culture of *S. aureus* ATCC 33591 was added to LB agar (45 °C) at a final concentration of 0.1% (vol/vol) and pour-plated into 90 mm petri dishes. Binding of **12** to purified lipid II was assessed using a spot-on-lawn assay. Premixed solutions (5 µL) containing analogue **12** (8 µg) with varying concentration of lipid II were spotted onto the agar, alongside individual controls of **12** and lipid II. After drying, plates were incubated overnight at 37 °C. This assay was performed in duplicates.

B: To assess the interaction between analogue **12** and lipid II, a culture of *S. aureus* ATCC 33591 was grown overnight in LB broth and inoculated into LB agar at a final concentration of 1% (v/v) at 45°C. This mixture was evenly distributed into 90mm petri dishes. Antibiotic inhibition haloes were previously determined by spotting analogue 12 and daptomycin (5 µL,8 µg/mL) onto agar plates and measuring the diameter of the halo after overnight incubation at 37 °C. The binding affinity of analogue **12** and lipid II was later examined by applying purified lipid II (300 µM, 2 µL) to the edge of the antibiotic halo. Analogue **12** and Daptomycin were applied to the inoculated agar plate at a spaced distance, once the antimicrobial solution drops had dried, lipid II was spotted on the edge of the inhibition halo. The plates were then incubated overnight at 37 °C. This assay was performed in duplicates.

XIII. References

(1) Karak, M.; Cloonan, C. R.; Baker, B. R.; Cochrane, R. V. K.; Cochrane, S. A. Optimizations of Lipid II Synthesis: An Essential Glycolipid Precursor in Bacterial Cell Wall Synthesis and a Validated Antibiotic Target. *Beilstein J. Org. Chem.* **2024**, *20* (1), 220–227.

(2) Shukla, R.; Peoples, A. J.; Ludwig, K. C.; Maity, S.; Derks, M. G. N.; De Benedetti, S.; Krueger, A. M.; Vermeulen, B. J. A.; Harbig, T.; Lavore, F.; Kumar, R.; Honorato, R. V.; Grein, F.; Nieselt, K.; Liu, Y.; Bonvin, A. M. J. J.; Baldus, M.; Kubitscheck, U.; Breukink, E.; Achorn, C.; Nitti, A.; Schwalen, C. J.; Spoering, A. L.; Ling, L. L.; Hughes, D.; Lelli, M.; Roos, W. H.; Lewis, K.; Schneider, T.; Weingarth, M. An Antibiotic from an Uncultured Bacterium Binds to an Immutable Target. *Cell* **2023**, *186* (19), 4059-4073.e27.

(3) Kazimierczuk, K.; Orekhov, V. Y. Accelerated NMR Spectroscopy by Using Compressed Sensing. *Angew. Chemie Int. Ed.* **2011**, *50* (24), 5556–5559.

(4) Parmar, A.; Iyer, A.; Prior, S. H.; Lloyd, D. G.; Leng Goh, E. T.; Vincent, C. S.; Palmai-Pallag, T.; Bachrati, C. Z.; Breukink, E.; Madder, A.; Lakshminarayanan, R.; Taylor, E. J.; Singh, I.

Teixobactin Analogues Reveal Enduracididine to Be Non-Essential for Highly Potent Antibacterial Activity and Lipid II Binding. *Chem. Sci.* **2017**, *8* (12), 8183–8192.

(5) Parmar, A.; Lakshminarayanan, R.; Iyer, A.; Mayandi, V.; Leng Goh, E. T.; Lloyd, D. G.; Chalasani, M. L. S.; Verma, N. K.; Prior, S. H.; Beuerman, R. W.; Madder, A.; Taylor, E. J.; Singh, I. Design and Syntheses of Highly Potent Teixobactin Analogues against Staphylococcus Aureus , Methicillin-Resistant Staphylococcus Aureus (MRSA), and Vancomycin-Resistant Enterococci (VRE) in Vitro and in Vivo. *J. Med. Chem.* **2018**, *61* (5), 2009–2017.

(6) Bann, S. J.; Ballantine, R. D.; McCallion, C. E.; Qian, P.-Y.; Li, Y.-X.; Cochrane, S. A. A Chemical-Intervention Strategy To Circumvent Peptide Hydrolysis by D -Stereoselective Peptidases. *J. Med. Chem.* **2019**, *62* (22), 10466–10472.

(7) Guo, L.; Kuipers, O. P.; Broos, J. An Engineered Nisin Analogue with a Hydrophobic Moiety Attached at Position 17 Selectively Inhibits Enterococcus Faecium Strains. *ACS Chem. Biol.* **2024**, *19* (9), 2023–2031.
